# Cloud point extraction of pyridine *N*-oxides using mixed micelles of PEGylated calix[4]pyrroles and non-ionic surfactants

**DOI:** 10.1039/d5ra08163g

**Published:** 2025-12-15

**Authors:** Julia Naulapää, Aliisa Kangas, Teppo O. Leino, Paula Perea Pérez, Kaisa Helttunen

**Affiliations:** a University of Jyväskylä, Department of Chemistry, Nanoscience Center Jyväskylä Finland kaisa.j.helttunen@jyu.fi

## Abstract

Pyridine *N*-oxides occur in pharmaceuticals and agrochemicals as solubilizing groups or as products of the oxidative metabolism of pyridine derivatives. Aryl-extended calix[4]pyrroles are supramolecular hosts that selectively bind pyridine *N*-oxides in host–guest complexes. A micelle-mediated extraction method was developed for the selective extraction of pyridine *N*-oxides from aqueous solutions using mixed micelles composed of aryl-extended calix[4]pyrroles and the non-ionic surfactants Triton X-100 and Tergitol 15-S-7. To this end, aryl-extended calix[4]pyrroles were functionalized with three different methoxy polyethylene glycol chains, and their binding constants for pyridine *N*-oxide and *p*-phenylpyridine *N*-oxide were determined with NMR titrations in DMSO-*d*_6_ and D_2_O. The effect of calix[4]pyrroles on the cloud point temperature of Triton X-100 was measured, and the hydrodynamic diameters of Triton X-100/calix[4]pyrrole mixed micelles were characterized using dynamic light scattering. The extraction efficiency of different calix[4]pyrrole/surfactant mixtures were evaluated. Notably, pyridine *N*-oxides showed no interaction with either Triton X-100 or Tergitol alone and at the investigated analyte concentration the calix[4]pyrrole does not reach cloud point, prompting the use of mixed micelles in which the calix[4]pyrrole binds pyridine *N*-oxide and the surfactant provides the clouding properties. The developed calix[4]pyrrole system represents the first example of cloud point extraction of pyridine *N*-oxides.

## Abstract 2

A novel water-soluble PEGylated calix[4]pyrrole binds pyridine *N*-oxide and enables its separation *via* cloud point extraction from water.

## Graphical abstract

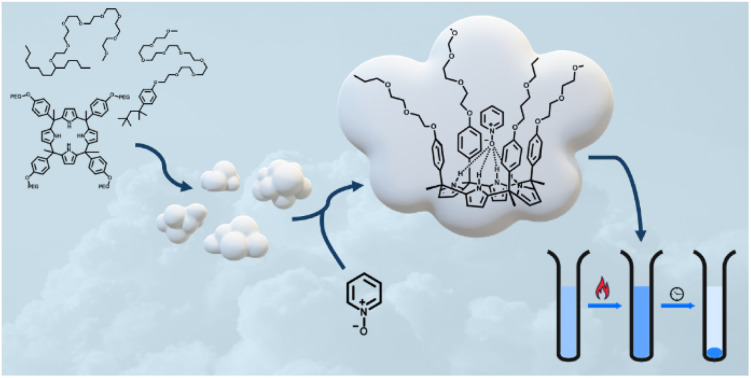

## Introduction

**Scheme 1:**
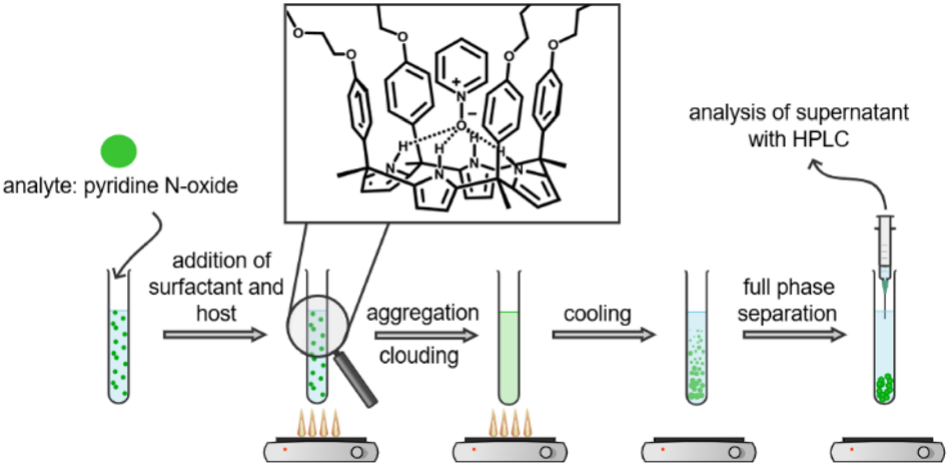
Cloud point extraction protocol and binding of pyridine *N*-oxide into a PEGylated calix[4]pyrrole. Diagram created with Chemix (2024). Retrieved from https://chemix.org.

Cloud point extraction (CPE, Scheme 1) is a separation method in which an analyte is captured from aqueous solution into a surfactant micelle and removed from the bulk solution into the coacervate phase, first introduced by Watanabe and Tanaka.^1^ CPE reduces the use of organic solvents in comparison to liquid–liquid extraction, aligning with the principle of green chemistry of using safer solvents and auxiliaries.^2^ High extraction efficiencies have been achieved by applying CPE for preconcentration of metal cations, such as Cr(iii) and Cr(vi).^3^ Organic ligands have been utilized to prepare hydrophobic metal complexes, for instance, pyridyl-azo compounds^3,4^ for complexation of Cr(iii), Cr(vi), Ga(iii) and In(iii), and O–O′-diethyl dithiophosphate for Cu(ii).^5^ CPE has also been developed for a variety of organic analytes, including toxic and harmful organic dyes,^6,7^ phenolic compounds,^8^ polyaromatic hydrocarbons, polychlorinated compounds, pesticides, and vitamins.^9^ CPE also shows potential for the recovery of valuable polyphenols from food waste.^10^

Triton X-100 (TX-100) is a non-ionic surfactant commonly used in CPE, and it has wide range of applications from biochemistry^11,12^ to industrial detergent and dispersant.^13^ However, TX-100 has safety problems, since it is a potential endocrine disruptor.^14^ Tergitol 15-S-7 (Tergitol) is a surfactant with a cloud point^15^ similar to that of TX-100 and is being considered a safer alternative^16^ due to the restrictions TX-100 is facing in the EU.

TX-100 and other surfactants form micelles at a concentration called the critical micelle concentration (CMC), above which the monomers of surfactant organize into spherical micelles held together by non-covalent forces.^17^ At cloud point the micelles aggregate leading to phase separation to supernatant and coacervate phases upon cooling.^18^ In general, cloud point temperature of the surfactant solution can be adjusted by the concentration of the surfactant,^19^ co-micellization with other surfactants,^20^ and presence of additives such as electrolytes,^21^ polyethylene glycols^19^ and alcohols,^19,22^ which have practical implications to the CPE method. Supramolecular hosts that bind metal analytes can also affect the cloud point temperature of the surfactant system.^23^ Supramolecular hosts, such as water-soluble calix[4]arenes, have also been successfully employed in CPE to selectively chelate Cu(ii) and La(iii) cations.^24^ A selective extraction method was developed by varying the extraction parameters, namely pH, equilibration and centrifugation times, and the concentrations of surfactant and chelating agent. So far, there has been a very limited number of applications of CPE enhanced by supramolecular host (host-assisted CPE) for organic analytes.^25^

Calix[4]pyrroles are a class of cup-shaped porphyrin derivatives, which can bind anionic and neutral polar guests into their cavity by hydrogen bonding.^26,27^ Due to their naturally poor water solubility the binding properties of calix[4]pyrroles have largely been investigated in organic solvents.^28–31^ In addition, water-soluble aryl-extended^32–34^ and super aryl-extended^35^ calix[4]pyrroles bearing carboxylate or pyridinium groups bind lactams and other cyclic carbonyl compounds^34^ and pyridine *N*-oxides^32,33,35^ in water, with high affinities being reported especially for pyridine *N*-oxides.

Pyridine *N*-oxides are important in medicinal chemistry as solubility improving functionalities^36^ and as synthetic intermediates for functionalized pyridines^37,38^ through activation at the 2-, 3-, and 6-positions of the pyridine ring.^39^ Pyridine *N*-oxides are formed by oxidation of pyridine, for example, in organic synthesis,^40^ and in the oxidative metabolism of pharmaceuticals^41^ and stimulants.^42^

Non-charged solubilizing groups, including polyethylene glycol (PEG), are regarded as biologically safe^43^ and have previously been attached to calix[*n*]arenes to solubilize them.^44^ Lately, the first non-ionic water-soluble calix[4]pyrrole was achieved by introduction of a PEG chain into one of the *meso*-positions located in the methyl bridge between pyrrole subunits in a hydroxypropyl-calix[4]pyrrole scaffold using Steglich esterification.^45^

This work presents the synthesis of PEGylated aryl-extended calix[4]pyrroles containing four PEG chains and demonstrates their application in host-assisted cloud point extraction of pyridine *N*-oxide (Scheme 1). The high water-solubility of pyridine *N*-oxide makes it an interesting organic analyte for CPE studies. The extraction efficiencies using traditionally utilized TX-100 and a safer alternative Tergitol as co-surfactants were compared. In addition, the effect of the PEG chain length to the solubility of the aryl-extended calix[4]pyrroles in water, as well as interactions with the non-ionic surfactants was investigated with dynamic light scattering.

## Results and discussion

### Synthesis

**Scheme 2:**
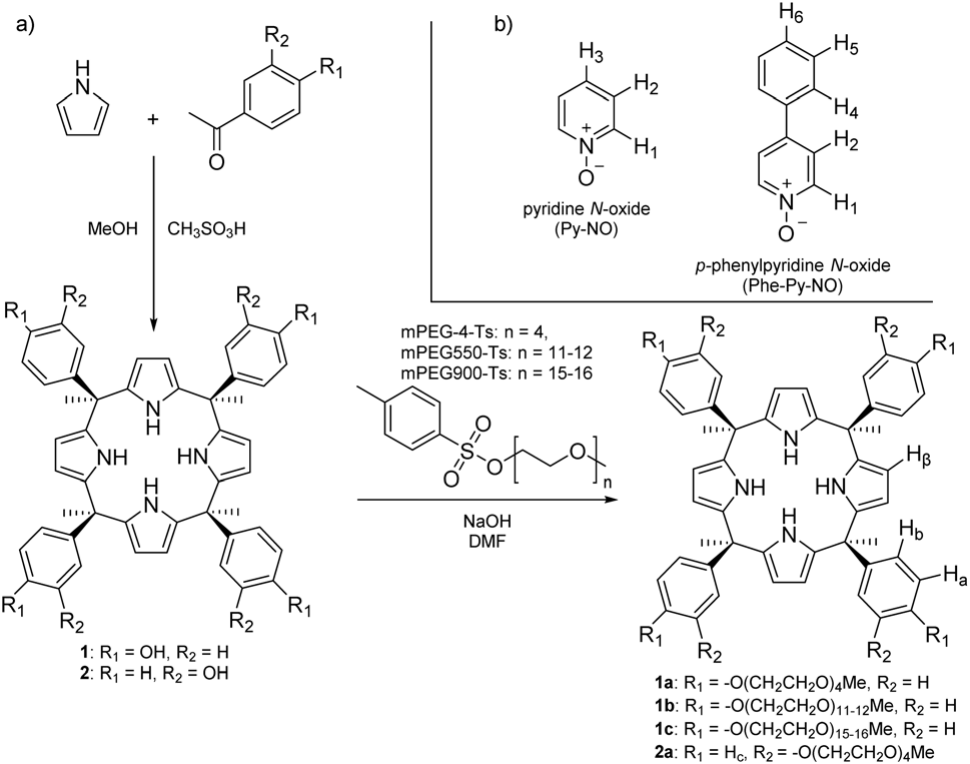
(a) Synthesis of PEGylated calix[4]pyrroles and (b) analytes pyridine *N*-oxide and *p*-phenylpyridine *N*-oxide.

To develop non-ionic water-soluble calix[4]pyrroles, aryl-extended αααα calix[4]pyrroles with *meso*-4-hydroxyphenyl (1) and *meso*-3-hydroxyphenyl groups (2) were first synthesized by modifying the protocols reported in the literature (Scheme 2a).^46–49^ The products were characterized with high resolution NMR and ESI-MS spectroscopy, which conformed with the expected values.

In the synthesis of compound 1, safer solvent options for the workup were tested. The synthesis of the aryl-extended calix[4]pyrrole yields a mixture of isomers αααα, αααβ, and ααββ.^47^ Previously, the isolation of these isomers from the reaction mixture has been achieved with chloroform^47,48^ or diethyl ether,^50,51^ both ranked as highly hazardous solvents.^52^ Instead of chloroform, less hazardous solvent dichloromethane was used to isolate the mixture of 1 and its isomers from the reaction mixture. 1 was separated from its isomers by heating the mixture in glacial acetic for a few minutes, further treating with hot 10% aqueous solution of acetic acid, and finally refluxing in 1 : 1 mixture of water and ethanol to remove the acetic acid from the cavity of 1. Compound 1 was obtained in 42% yield, which was in line with the 39% yield reported by Bonomo *et al.*^46^ and 45% yield reported by Jain and Mandalia in conventional reflux.††Microwave-assisted synthesis can further improve the yield by 20% as reported by Jain and Mandalia.^50^^50^ In the new workup method, the additional washes with *n*-hexanes after the crystallization from acetic acid^46^ can be avoided. Furthermore, we noticed that the exposure of 1 to 10% aqueous solution of acetic acid decreases the solubility of 1 to ethanol–water mixtures, compared to the use of glacial acetic acid alone, and thus less product is lost in the final purification step using ethanol and water.

Compounds 1 and 2 were PEGylated with a nucleophilic substitution reaction using NaOH as a base and tosylated monodisperse m-PEG4 (Scheme 2a). The functionalization of all OH groups was verified by the complete disappearance of the ^1^H NMR signal arising from the OH groups of 1 and 2 at 9.0–9.2 ppm, and ratio of the integral values of the β-pyrrolic protons at 5.9 ppm and CH_2_ protons at 3.7 and 4.0 ppm in the PEGylated calix[4]pyrrole derivatives 1a and 2a (Fig. S16). ESI-MS of the monodisperse PEGylated calix[4]pyrrole 1a showed the formation of a base peak from the chloride adduct under negative polarization and potassium adduct under positive polarization.

*Para*-substituted calix[4]pyrrole 1 was chosen for functionalization with polydisperse PEG-900 and PEG-550 (Scheme 2a) and subsequent studies due to the more environmentally friendly work-up protocol discussed above, and slightly higher binding affinity of the *para* substituted derivative 1a with the investigated analyte (pyridine *N*-oxide, *vide infra*). The polydisperse calix[4]pyrroles 1b and 1c were prepared with the same method as short chain PEG-derivatives (1a and 2a) and characterized with NMR spectroscopy and ATR-IR, where their spectra showed strong peaks at 2800 cm^−1^ attributed to the methylene C–H stretching of the PEG-chains. Intense signals at the same wavenumber were observed for the starting material, m-PEG550-Ts. Additionally, 1b and 1c showed broad peaks at 3500 cm^−1^ attributed to the NH stretching arising from the calix[4]pyrrole scaffold.

### Solubility and micellization

The solubility of the PEGylated calix[4]pyrroles in water was investigated to assess their suitability to applications in water. Whereas 1, 1a, 2 and 2a are soluble in methanol and DMSO but not in water, 1b and 1c are miscible with water. This allows their use in aqueous applications.

The CMC of 1b and 1c was determined with dynamic light scattering (DLS). The CMC of 1c was determined to be 0.16 mM. This value is lower than the CMC of *tert*-butyl calixarene-based PEGylated surfactants with similar molecular weights,^53^ suggesting that PEGylated calix[4]pyrroles are more hydrophobic. For 1b the CMC is significantly lower than for 1c, 3.4 µM. The trend is in line with the observations for PEGylated calixarenes,^53^ where the CMC was lower for shorter PEG chain lengths due to the increased aggregation of more hydrophobic amphiphiles.

The interactions between non-ionic surfactant Triton X-100 and aryl-extended calix[4]pyrroles 1, 2, as well as PEGylated calix[4]pyrroles 1a, 2a, 1b and 1c were investigated to develop a mixed-micelle systems suitable for cloud point extraction. Introduction of 1 w/v% TX-100 into the calix[4]pyrrole solution solubilizes all investigated calix[4]pyrroles in 3% aqueous methanol, whereas without TX-100 compounds 1, 2, 1a and 2a are not soluble in 3% MeOH. In addition, 1, 2, 1a and 2a are not solubilized in water by TX-100, limiting their use in CPE.

**Fig. 1:**
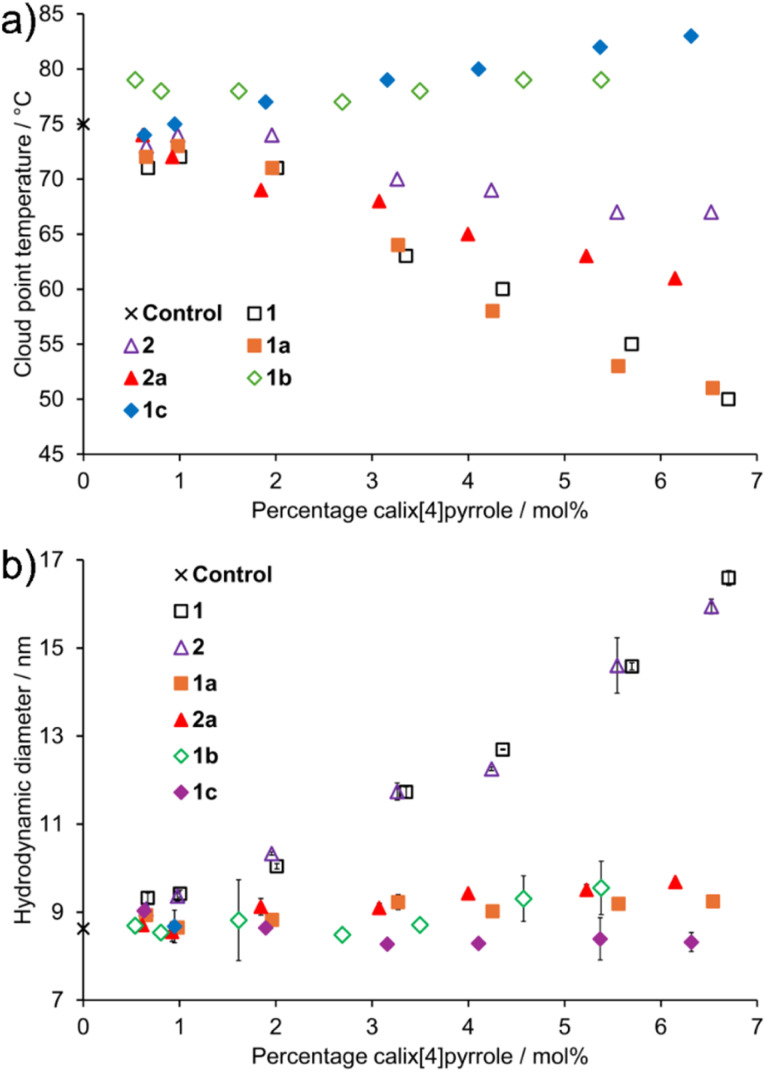
(a) Cloud point temperatures (error ±1 °C) and (b) hydrodynamic diameters (*Z*-average) of TX-100 with different ratios of calix[4]pyrroles 1–6 in 3% methanol solution, error as standard deviation of 3 measurements.

Samples containing 1 w/v% TX-100 in 3% methanol with varying molar percentages (0–7 mol%) of 1, 2, 1a, 2a, 1b and 1c were made. The solutions were heated to observe the cloud point temperature (Fig. 1a), and their hydrodynamic diameters were measured with DLS (Fig. 1b). The addition of 6 mol% of water-soluble calix[4]pyrroles 1b and 1c to TX-100 increased the cloud point temperature of the solution by 3 °C and 7 °C, respectively. In contrast, the addition of water-insoluble calix[4]pyrroles 1 and 1a caused a 25 °C decrease in the cloud point temperature. Calix[4]pyrrole derivatives bearing a 3-hydroxyphenyl substituent, 2 and 2a, induced a more moderate decrease in the cloud point temperature by 8–14 °C. The results indicate that the TX-100 system tolerates higher loading of more hydrophilic PEGylated calix[4]pyrroles before reaching cloud point at room temperature, which would be undesirable for the extraction. On the other hand, water-soluble 1b and 1c do not substantially increase the cloud point of TX-100 rendering the systems applicable for CPE.

Dynamic light scattering was used to characterize the changes induced by the calix[4]pyrroles to the micelle size. The 1% solution of TX-100 in 3% aqueous methanol contained micelles with a hydrodynamic diameter (*Z*-average) of 8.6 nm, characteristic size of TX-100 micelles in water (Fig. 1b).^54^ The addition of 1 and 2 to TX-100 solution resulted in an increase in the hydrodynamic diameter of the micelles, from 9.4 nm to approximately 18 nm at 7 mol% of calix[4]pyrrole. The addition of calix[4]pyrroles with short (1a, 2a) and long (1b, 1c) PEG-chains resulted in less substantial change in the size of the micelles. However, when calix[4]pyrroles were added, the standard deviation of *Z*-average sizes increased for some data points. This was taken as an indication that the samples were less monodisperse, containing potentially more aggregates that appeared in front of the laser occasionally during the measurements. The hydrophobicity of the additives affect the interaction with the TX-100 micelles, since more hydrophobic phenolic compounds have been shown to increase the size of TX-100 micelles by entering the micellar core, whereas hydrophilic phenolic compounds do not affect the size.^13^ These results suggest that 1 and 2, which are more hydrophobic, incorporate the micellar core, whereas the more hydrophilic PEGylated calix[4]pyrroles reside in the outer layer or on the surface of the TX-100 micelles when added in less than 10 mol%. To probe interactions between 1c and Triton X-100, NOESY NMR was recorded in D_2_O at 1 : 1.3 ratio of the components, respectively. A NOE correlation was observed between the CH_3_ protons at the lipophilic part of Triton X-100 and H_β_ and *meso*-CH_3_ protons of the calix[4]pyrrole 1c outside of the binding cavity (Scheme 2; Fig. S41 and S42). This indicates that the two components are in close proximity, most likely forming mixed micelles.

### Host–guest complexation

**Fig. 2:**
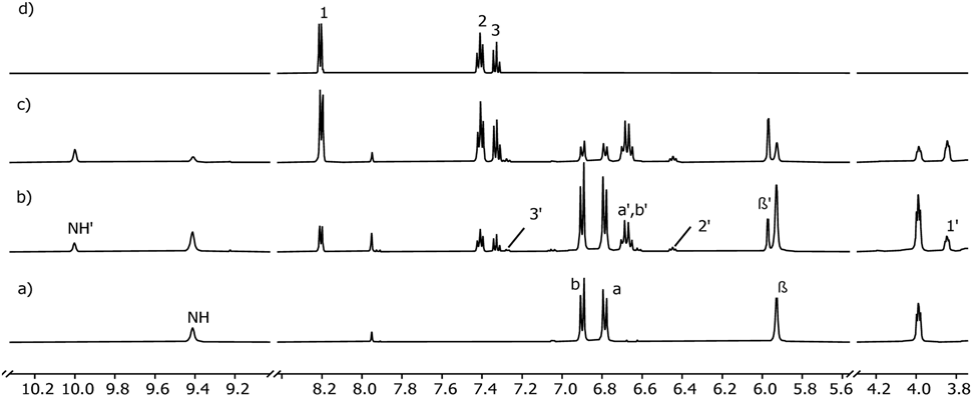
Selected region of the ^1^H NMR (500 MHz, DMSO-*d*_6_, 298 K) spectra from the titration of 1a with Py-NO: (a) 1a; (b) 1a + Py-NO (1 : 1 molar ratio); (c) 1a + Py-NO (1 : 5.6 molar ratio); (d) Py-NO. Primed letters are assigned to the proton signals of the complex.

Pyridine *N*-oxide (Py-NO) and *p*-phenylpyridine *N*-oxide (Phe-Py-NO, Scheme 2b) are known to bind strongly to the cavity of various aryl-extended calix[4]pyrroles^31^ forming host–guest inclusion complexes in D_2_O and CD_3_CN with *K*_a_ = 0.2–2 × 10^4^ M^−1^.^33^ Therefore, these compounds were selected for development of selective cloud point extraction systems with calix[4]pyrroles. The binding of Py-NO and Phe-Py-NO with 1a, 1b and 1c was investigated with NMR titrations at 4–5 mM host concentration. In addition, complexation of Py-NO was studied with 2a. Titrations of 1a, 2a and 1b were performed in DMSO-*d*_6_. Upon incremental addition of Py-NO to the 1a a new set of signals was observed (Fig. 2). A new signal at 10 ppm was assigned as a signal arising from NH protons of the 1 : 1 host–guest complex 1a⊃Py-NO, hydrogen bonding with the *N*-oxide group included in the calix[4]pyrrole cavity. Alongside the emergence of the new NH signal, a gradual decrease of the NH signal for the free host at 9 ppm was observed as an indication of slow exchange dynamics between free and bound species relative to the NMR timescale suggesting high kinetic stability for the complex. Guest protons shifted upfield, with the proton adjacent to the *N*-oxide moiety (H_1_) moving from 8.2 ppm to 3.8 ppm, which indicates shielding upon inclusion to the aromatic cavity.

However, after addition of 5–6 equivalents of the guest the spectrum still showed signals from the free host. Thus, in these conditions, the binding constant calculated from the integral values of the free and bound host was low, in the order of 100 M^−1^. The relatively low binding constants suggest that DMSO competes with the guest binding or solvates the host and guest very efficiently.^55^

Similar results were obtained, when 1a was titrated with Phe-Py-NO, and 1b was titrated with Py-NO and Phe-Py-NO (Table 1). Titration of 2a and Py-NO showed similar changes in the NH proton signals, however, a smaller binding constant was calculated. Due to the lower binding affinity in these conditions, this host was not studied further.

**Table 1:** Binding constants (*K*_a_) for host–guest complexes estimated from NMR titrations (M^−1^). Values are average of two measurements and their standard deviation

Host	Py-NO^a^	Phe-Py-NO^a^	Py-NO^b^	Phe-Py-NO^b^
1a	77 ± 42	151 ± 45	—c	—c
2a	13 ± 13	—c	—c	—c
1b	97 ± 66	121 ± 2	—c	—c
1c	—c	—c	>10^4^	>10^4^

a In DMSO-*d*_6_.

b In D_2_O.

c Not determined.

**Fig. 3:**
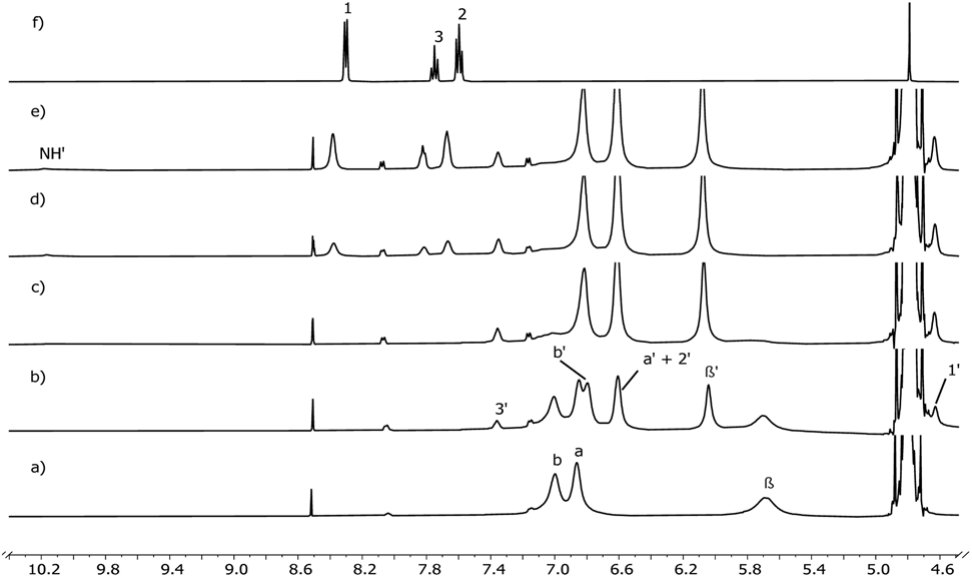
Selected region of the ^1^H NMR (500 MHz, D_2_O, 298 K) spectra from the titration of 1c with Py-NO: (a) 1c; (b) 1c + Py-NO (1 : 0.3 molar ratio); (c) 1c + Py-NO (1 : 0.5 molar ratio); (d) 1c + Py-NO (1 : 0.8 molar ratio); (e) 1c + Py-NO (1 : 1.1 molar ratio); (f) Py-NO. Primed letters are assigned to the proton signals of the complex.

The solubility of 1c in water allowed binding studies in D_2_O with both Py-NOs at 3–8 mM concentration of the host, a value that is above the CMC. The signals of the host were significantly broadened in D_2_O in comparison to DMSO-*d*_6_ (Fig. 3), which hindered accurate integration. Initially, the signal arising from the NH protons of the free host was not visible, potentially due to hydrogen bonds with the solvent molecules forming and breaking at an intermediate NMR timescale. Upon addition of Py-NO, the NH-peak emerged at ∼10 ppm, at a similar position as in the experiments in DMSO-*d*_6_. In addition, the pyrrole β-position proton resonance at 5.7 ppm diminished and a new peak at 6.00 was observed. When approximately 0.5 equivalents of the guest had been added according to the calculated concentrations, the original β-pyrrolic proton peak had disappeared. A similar change was seen in the CH_3_ peak at 1.9 ppm which reduced to zero alongside the emergence of a new peak at 1.8 ppm (Fig. S33). Simultaneously, signal of bound guest proton H3′ showed approximately 1 : 1 ratio with the host. Further additions of the guest made the free guest signals visible, slightly broadened and downfield-shifted relative to those of pure Py-NO in D_2_O. Since all added Py-NO was complexed below addition of 1 equivalent, it indicates that a thermodynamically stable host–guest complex was formed with a *K*_a_ > 10^4^ M^−1^. As expected, high affinity was observed when Phe-Py-NO was used as a guest. Both 1c⊃Py-NO and 1c⊃Phe-Py-NO complexes remained fully water-soluble.

### Cloud point extraction of Py-NO with 1c

Due to its water-solubility and formation of 1c⊃Py-NO inclusion complex, 1c was chosen as the ligand to host-assisted CPE of Py-NO with non-ionic surfactants. Py-NO in water was mixed with 1c and TX-100 or Tergitol, and clouding was triggered by heating the solution (Scheme 1). After phase separation, the supernatant was separated from the surfactant rich coacervate phase and analysed with reverse phase HPLC with UV-vis detection at 280 nm.

**Fig. 4:**
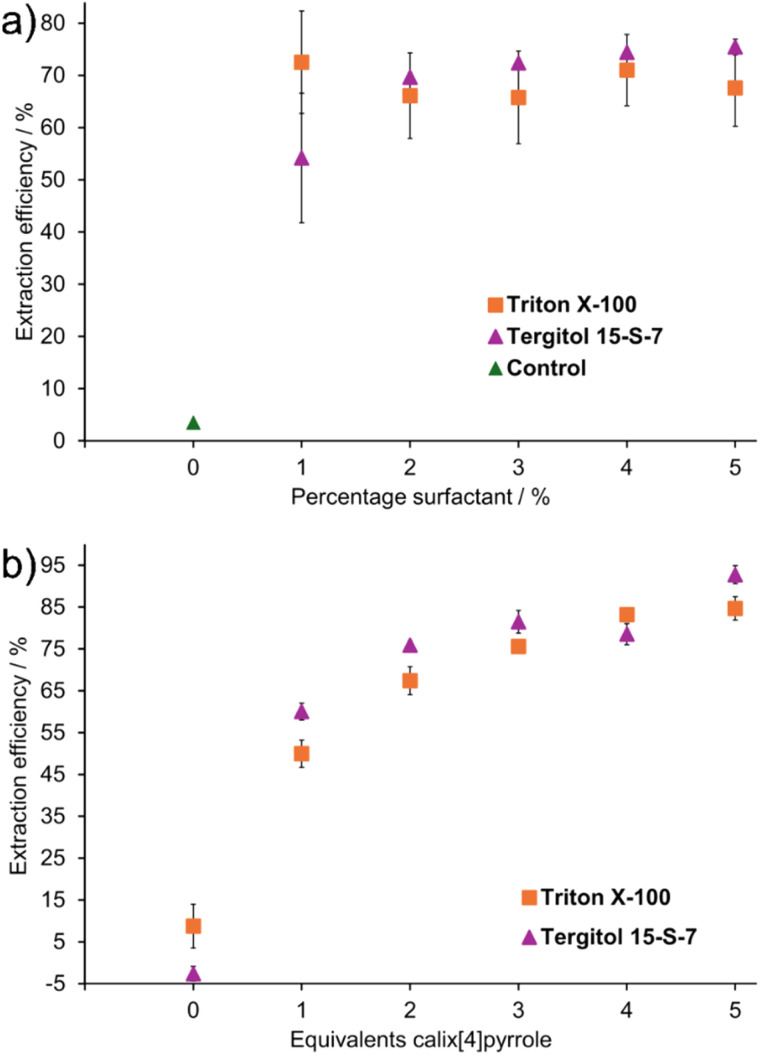
Extraction efficiency of cloud point extraction of 0.4 mM Py-NO with (a) 2 molar equivalents of 1c with 0–5 percentages of TX-100 or Tergitol (error bar is shown as standard deviation from three replicates) and (b) 2.8% TX-100 or 2.7% Tergitol with 0–5 molar equivalents of 1c.

Typically, the optimal surfactant concentration in CPE is near 1–3% and therefore, effect of surfactant concentration for extraction was tested.^8,23^ Varying the concentration of TX-100 or Tergitol between 1–5% had only a modest effect on the CPE efficiency at 0.4 mM concentration of Py-NO (Fig. 4a), when 2 molar equivalents of the host was used. The best extraction efficiency of 75.4 ± 1.5% was obtained at 5% concentration of Tergitol and similar 72.35 ± 0.01% efficiency was achieved at 3% Tergitol, whereas only 54.2 ± 12.4% of Py-NO was extracted at 1% Tergitol. As a control, CPE was also attempted without surfactant using 2 equivalents of 1c, which produced only 3.5% extraction efficiency (Fig. 4a and Table S13). Without surfactant, 1c does not reach cloud point at this concentration and no phase separation takes place, which explains the low extraction efficiency. Surfactant concentration of 2.8% was chosen for further studies.

The relative concentration of the calix[4]pyrrole 1c against 0.4 mM Py-NO had a large effect on the extraction efficiency (Fig. 4b). In control experiments without 1c, Py-NO was not extracted to 2.8% TX-100 or Tergitol solutions (CPE efficiency max. 9%; Tables S14 and S16). When 1 molar equivalent of 1c was added, 50–60% extraction efficiency was obtained for TX-100 or Tergitol. By increasing host concentration to 5 equivalents (2.0 mM 1c), the extraction efficiency increased up to 84.7 ± 2.8% in Triton X-100 and 92.7 ± 2.2% in Tergitol (Fig. 4b).

The poor extraction efficiency with Tergitol and TX-100 without 1c (Fig. 4b) can be explained by lack of intermolecular interactions between the surfactant and Py-NO based on ^1^H NMR titrations in D_2_O (Fig. S35 and S36). Thus, the cloud point extraction of Py-NO with TX-100 or Tergitol requires a specific binding site provided by a supramolecular host such as 1c to be efficient. The calix[4]pyrrole binds pyridine *N*-oxide in a host–guest complex, and surfactant (TX-100 or Tergitol) enables extraction of the complex at low concentration of 1c. Comparison of the TX-100 and Tergitol as a co-surfactant showed that TX-100 had a higher extraction efficiency below 2% surfactant concentration with 0.8 mM of 1c than Tergitol, but with higher surfactant or host concentrations Tergitol performed slightly better.

## Experimental

### General

Pyrrole (from TCI Europe) was distilled prior to use. Other solvents and reagents, including *p*-phenylpyridine *N*-oxide (from TCI Europe), 3-hydroxyacetophenone and dry DMF (from Thermo-Scientific), polyethylene glycol methyl ether tosylate (m-PEG900-Ts, from Aldrich), polyethylene glycol methyl ether (m-PEG550), Triton X-100, methanesulfonic acid, and pyridine *N-*oxide (from Sigma-Aldrich), and 4-hydroxyacetophenone (from Merck) were used without further purification. Aa-grade ethanol was used in recrystallizations. Purified water (Millipore Elix 3 UV system equipped with a Progard 2 pretreatment pack) was used in syntheses and micellar studies.

### Synthesis

#### *Meso*-4-hydroxyphenyl calix[4]pyrrole (1)

Calix[4]pyrrole 1 was prepared according to known procedures^46–48^ with slight modifications. Methanesulfonic acid (0.60 ml, 9.2 mmol) was added dropwise over 5 min to a solution of 4-hydroxyacetophenone (2.61 g, 19.0 mmol) and freshly distilled pyrrole (1.33 ml, 19.0 mmol) in anhydrous methanol (56 ml) under nitrogen atmosphere. The reaction mixture was stirred at room temperature for 24 h and quenched with triethylamine (1.28 ml). The resulting mixture was diluted with dichloromethane (56 ml) and filtered through a short pad of SiO_2_ using DCM/MeOH (1 : 1) eluent to remove polymeric by-products. Volatiles were removed under reduced pressure in a rotary evaporator to give a dark red solid. Glacial acetic acid (15 ml) was added to the residue, and the resulting suspension was heated at 105 °C for 15 min. The suspension was allowed to cool down to room temperature and the solid was collected by filtration, washed with glacial acetic acid, and dried under reduced pressure in a vacuum line. The resulting pale pink solid was suspended in a 10% aqueous solution of acetic acid (30 ml) and refluxed for 15 min. The mixture was cooled down to room temperature, and the volatiles were removed under reduced pressure in a rotary evaporator. The resulting solid was suspended in a mixture of EtOH and H_2_O (1 : 1, 20 ml), refluxed for 15 min, allowed to cool down to room temperature, and the solid was collected by filtration. The solid was washed with a solution of EtOH and H_2_O (1 : 1) and dried under reduced pressure in a vacuum line to give EtOH solvate of αααα-isomer of 1 (1 : 1) as a pale pink solid (1.56 g, 42%).

^1^H NMR (DMSO-*d*_6_, 500 MHz) *δ* (ppm) 1.71 (s, 12H, CH_3_), 5.93 (d, *J* = 2.6 Hz, 8H), 6.62 (d, *J* = 8.4 Hz, 8H), 6.68 (d, *J* = 8.4 Hz, 8H), 9.26 (s, 4H), 9.43 (s, 4H).

^13^C NMR (DMSO-*d*_6_, 126 MHz) *δ* (ppm) 31.6, 43.4, 104.2, 114.9, 127.9, 137.5, 140.5, 155.9.

HRMS (ESI-Q-TOF) *m/z*: calcd for C_48_H_45_N_4_O_4_^+^ 741.3435; found 741.3419.

#### *Meso*-3-hydroxyphenyl calix[4]pyrrole (2)

Calix[4]pyrrole 2 was prepared according to known procedure^49^ with slight modifications. 3-Hydroxyacetophenone (2.46 g, 18.1 mmol) and freshly distilled pyrrole (1.3 ml, 18.7 mmol) were dissolved into 30 ml anhydrous methanol and cooled with ice. Methanesulfonic acid (0.25 ml, 3.9 mmol) was dissolved into 15 ml anhydrous methanol and added dropwise over 5 min to the reaction, after which the reaction mixture was allowed to warm to room temperature. The reaction mixture was stirred at room temperature overnight and quenched with triethylamine (0.5 ml) dissolved into 50 ml water. The resulting brown residue was moved to another flask and diethyl ether was added. The mixture was sonicated and filtered. Solvent was evaporated under reduced pressure in a rotary evaporator to give a brown sticky residue. Residue was recrystallized from hot acetonitrile to give 2 αααα-isomer as a white powder (143 mg, 4%).

^1^H NMR (500 MHz DMSO-*d*_6_) *δ* (ppm) 1.76 (s, 12H), 5.97 (d, *J* = 2.5 Hz, 8H), 6.38–6.41 (m, 8H), 6.45 (dd, *J* = 8.0, 1.4 Hz, 4H), 6.98 (t, *J* = 8.0 Hz, 4H), 9.09 (s, 4H), 9.47 (s, 4H).

^13^C (126 MHz DMSO-*d*_6_) *δ* (ppm) 31.3, 44.1, 104.2, 113.2, 114.0, 117.9, 129.1, 137.3, 151.8, 157.4.

HRMS (ESI-Q-TOF), *m/z*: [M + H]^+^ calcd for C_48_H_45_N_4_O_4_^+^ 741.3435; found 741.3414.

#### Tosylated polyethylene glycol methyl ether (m-PEG4-Ts)

m-PEG4-OH was tosylated according to a modified literature procedure.^56^ m-PEG4-OH (5 ml, 5.41 g, 25.96 mmol) was dissolved into 7 ml THF and the solution was cooled with ice under a N_2_ atmosphere. NaOH (1.78 g, 44.58 mmol) dissolved into 9 ml of deionized water was added dropwise. *p*-Toluenesulfonyl chloride (5.41 g, 28.36 mmol) dissolved into 9 ml of deionized water was added dropwise. The reaction mixture was allowed to warm up to room temperature, and the solution was stirred for 19 minutes at room temperature. The reaction mixture was diluted with 70 ml of diethyl ether and washed three times with 1M NaOH (10 ml), two times with deionized water (20 ml) and once with brine (10 ml). The organic phase was dried over Na_2_SO_4_, filtrated and the solvent was evaporated under reduced pressure in a rotary evaporator to give m-PEG4-Ts as a clear viscous liquid (7.23 g, 78%).

^1^H NMR (MeOD, 500 MHz) *δ* (ppm) 2.45 (s, 3H), 3.35 (s, 3H), 3.52–3.56 (m, 6H), 3.59–3.62 (m, 6H), 3.65 (m, 2H), 4.14 (m, 2H), 7.44 (d, *J* = 8.0, 2H), 7.80 (d, *J* = 8.0 Hz, 2H).

^13^C NMR (MeOD, 126 MHz) *δ* (ppm) 21.6, 59.1, 69.7, 70.9, 71.3, 71.5, 72.9, 129.0, 131.1, 134.4, 146.4.

HRMS (ESI-Q-TOF) *m/z*: [M + Na]^+^ calcd for C_16_H_26_O_7_SNa^+^ 385.12915; found 385.1302, [M + K]^+^ calcd. for C_16_H_26_O_7_SK^+^ 401.10308; found 401.1029.

#### Tosylated polyethylene glycol methyl ether (m-PEG550-Ts)

m-PEG550-OH was tosylated according to a known procedure.^57^ m-PEG550-OH (2.15 g, 3.9 mmol) dissolved into 2.2 ml anhydrous pyridine was cooled on ice and stirred for 10 minutes. *p*-Toluenesulfonyl chloride (0.99 g, 5.2 mmol) was dissolved in 1.5 ml anhydrous pyridine and added dropwise to the reaction mixture using a syringe and the mixture was stirred on ice for 6 hours. The reaction was quenched by adding a small amount of pure ice into the reaction flask, followed by the slow addition of 18 ml 6 N HCl solution while continuously stirring. The mixture was extracted three times with DCM (20 ml), and the combined organic layers were washed with 2 N HCl solution (60 ml). The organic layer was collected, dried over an anhydrous Na_2_SO_4_. Solvent was evaporated under reduced pressure in a rotary evaporator to give m-PEG550-Ts as a slightly yellow, clear oil (2.62 g, 95%).

^1^H NMR (DMSO-*d*_6_, 500 MHz) *δ* (ppm): 2.42 (s, 3H), 3.24 (s, 3H), 3.42–3.44 (m, 6H), 3.48–3.58 (m, 32H), 3.57 (t, *J* = 5.0 Hz, 2H), 4.11 (t, *J* = 5.0 Hz, 2H), 7.48 (d, *J* = 8.0 Hz, 2H), 7.78 (d, *J* = 8.0 Hz, 2H).

^13^C NMR (DMSO-*d*_6_, 126 MHz) *δ* (ppm) 21.1, 58.1, 67.9, 70.0, 71.3, 72.0, 127.6, 130.1, 132.4, 144.9.

#### General procedure for PEGylated calix[4]pyrroles

Calix[4]pyrrole 1 or 2 (120 mmol) and NaOH (12 eq.) were dissolved into 3.4 ml of dry DMF and stirred at room temperature under a N_2_ atmosphere for 30 minutes. Polyethylene glycol methyl ether tosylate (4.5–5 eq.) was dissolved into 3.6 ml of dry DMF and added to the mixture which was refluxed at 80 °C overnight. The liquid was pipetted into another flask to separate from solid NaOH. The solvent was co-evaporated with toluene under reduced pressure to remove DMF. The PEGylated calix[4]pyrroles were further purified either by flash chromatography or manual column chromatography.

#### PEG4-ylated *meso*-4-hydroxyphenyl calix[4]pyrrole (1a)

The product was purified by flash column chromatography (0 to 10% methanol in DCM) using RediSep Gold silica column and dried in vacuum. Yield of dark brown wax 77%. *R*_F_ (10% MeOH in DCM) 0.33.

^1^H NMR (500 MHz DMSO-*d*_6_) *δ* 1.74 (s, 12H, 3.23 (s, 12H, 3.41–3.43 (m, 12H), 3.49–3.55 (m, 48H), 3.67 (t, *J* = 4.8 Hz, 8H), 3.99 (t, *J* = 4.8 Hz, 8H), 5.93 (s, 8H), 6.78 (d, *J* = 8.7 Hz, 8H), 6.90 (d, *J* = 8.9 Hz, 8H), 9.42 (s, 4H).

^13^C (126 MHz DMSO-*d*_6_) *δ* 31.3, 43.4, 58.1, 67.2, 68.8, 69.6, 69.8, 69.9, 71.3, 104.5, 114.3, 127.9, 137.4, 142.2, 157.2.

HRMS (ESI-Q-TOF) *m/z*: calcd. [M + Cl]^+^ for C_84_H_116_N_4_O_20_Cl^−^ 1535.78714; found 1535.7887.

#### PEG4-ylated *meso*-3-hydroxyphenyl calix[4]pyrrole (2a)

The product was purified by flash column chromatography (0 to 10% methanol in DCM) using RediSep Gold silica column and dried in vacuum. Yield of yellow oil 65%. *R*_F_ (10% MeOH in DCM) 0.31.

^1^H NMR (500 MHz DMSO-*d*_6_) *δ* (ppm) 1.79 (s, 12H), 3.22 (s, 12H), 3.39–3.44 (m, 18H), 3.46–3.52 (m, 52H), 3.53–3.55 (m, 8H), 3.58–3.60 (m, 8H), 3.74 (t, *J* = 5.0, 8H), 3.98 (t, *J* = 5.0, 8H), 5.98 (s, 8H), 6.43–6.47 (m, 8H), 6.68 (dd, ^3^*J* = 8.2 Hz, 2.2 Hz, 4H), 7.18 (t, *J* = 8.0 Hz, 4H), 9.46 (s, 4H).

^13^C NMR (DMSO- *d*_6_, 126 MHz) *δ* (ppm): 31.1, 44.1, 58.1, 66.9, 69.0, 69.6, 69.8, 70.0, 71.3, 72.4, 104.7, 111.4, 113.9, 119.1, 129.3, 137.0, 151.9, 158.6.

HRMS (ESI-Q-TOF) *m/z*: [M + H]^+^ calcd for C_84_H_117_N_4_O_20_^+^ 1501.82612; found 1501.8216, [M + K]^+^ calcd for C_84_H_116_N_4_O_20_K^+^ 1539.78145; found 1539.7795.

#### PEG550-ylated *meso*-4-hydroxyphenyl calix[4]pyrrole (1b)

The product was purified using manual column purification (10% MeOH in DCM; *R*_F_ 0.28). The product was dissolved in DCM and sonicated in the presence of cellulose. The cellulose was removed, and the solution was washed with 1M HCl.^58^ Organic phase was dried with Na_2_SO_4,_ and solvent was evaporated under reduced pressure with rotary evaporator. Yield of yellow oil 29%.

^1^H NMR (DMSO- *d*_6_, 500 MHz) *δ* (ppm): 1.74 (s, 12H), 3.23 (s, 12H), 3.42 (t, *J* = 4.8, 12H), 3.48–3.54 (m, 250H), 3.67 (t, *J* = 8.5 Hz, 8H), 3.99 (t, *J* = 8.5 Hz, 8H), 5.92 (s, 8H), 6.78 (d, *J* = 8.7 Hz, 8H), 6.90 (d, *J* = 8.8 Hz, 8H), 9.42 (s, 4H).

^13^C NMR (DMSO- *d*_6_, 126 MHz) *δ* (ppm): 31.2, 43.4, 58.1, 68.8, 69.6, 69.8, 71.3, 104.5, 114.3, 127.9, 137.4, 142.2, 157.2.

#### PEG900-ylated *meso*-4-hydroxyphenyl calix[4]pyrrole (1c)

The product was purified by manual flash column chromatography (10% MeOH in DCM; *R*_F_ 0.29). Solvent was evaporated under reduced pressure with a rotary evaporator. The residue was dissolved into DCM and washed with 1M HCl. Yield of yellow wax 70%.

^1^H NMR (500 MHz DMSO-*d*_6_) *δ* 1.75 (s, 12H), 3.24 (s, 12H), 3.42 (t, *J* = 4.5, 8H), 3.41–3.43 (m, 12H), 3.50–3.56 (m, 370H), 3.67 (t, *J* = 4.5, 8H), 3.99 (t, *J* = 4.5, 8H), 5.92 (s, 8H), 6.79 (d, *J* = 8.5, 8H), 6.90 (d, *J* = 8.5, 8H), 9.42 (s, 4H).

^13^C (126 MHz DMSO-*d*_6_) *δ* 31.2, 43.4, 58.0, 67.2, 68.8, 69.6, 69.8, 71.3, 104.5, 114.3, 127.9, 137.3, 142.2, 157.2.

### NMR titration

The host solution was prepared by dissolving the corresponding calix[4]pyrrole or surfactant (TX-100 or Tergitol) into DMSO-*d*_6_ or D_2_O. The guest solution was prepared by dissolving the guest (Py-NO or Phe-Py-NO) into 1 ml of the prepared host solution. Aliquots of the guest solution were added incrementally to the host solution, and in between the additions ^1^H NMR spectra were recorded. Residual DMF or DCM from the synthesis was used as an internal standard in calibrating the integrals. The complexation between all the calix[4]pyrrole hosts and the guests displayed slow exchange dynamics based on the gradual disappearance of the peaks assigned to the free host alongside the emergence of a new set of peaks assigned as bound host.

The binding constant between hosts 1a, 1b and 2a with Py-NO and Phe-Py-NO were calculated^59,60^ according to eqn (1) and (2), from the integrals of NH in the complex (*n*) and free host (m), and concentrations of host and guest before the equilibration ([*H*_0_] and [*G*_0_], respectively) at approximately 1 : 1 ratio of guest to host.1
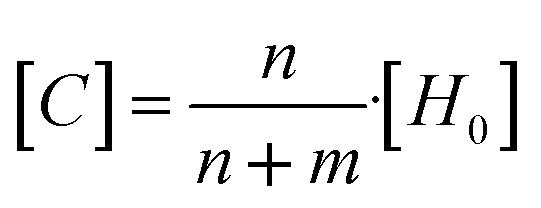
2
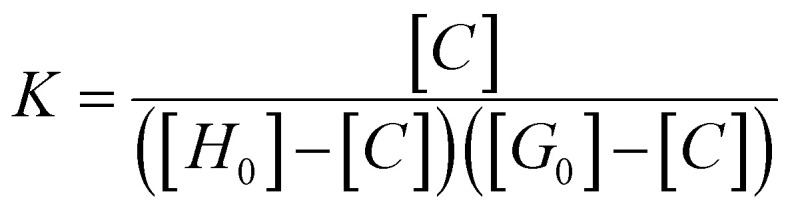
In addition, for each host–guest pair, one sample with 1 : 1 ratio of calix[4]pyrrole and guest was made. The average binding constant and standard deviation was calculated.

### Micellar studies

The co-micellization of 1, 1a, 2, 2a, 1b and 1c with the surfactant TX-100 were investigated by cloud point temperature observations and micelle size measurements with dynamic light scattering. Stock solutions of the calix[4]pyrroles were prepared in methanol and the surfactant into Millipore water. Samples were prepared by mixing different ratios of the calix[4]pyrrole stock solutions with the TX-100 stock solution, keeping the surfactant concentration constant at 0.97 w/v% and methanol concentration at 3.3%. The cloud point temperature of the samples was determined by heating 3.1 ml of the sample in a 4 ml glass vial sealed with a screw cap. The cloud point temperature of the solutions was determined by heating the samples incrementally by 1 °C in a block heater followed by a 3-minute equilibration period at each temperature, after which each sample was visually observed. The cloud point temperature was recorded to be the temperature, where turbidity was observed.

The particle sizes of the TX-100/calix[4]pyrrole mixed micelles were determined by measuring the hydrodynamic diameters with DLS at 25 °C. Each sample was measured as a triplicate, and average value for the Zeta-average and polydispersity index are reported.

### Cloud point extraction of pyridine *N*-oxide

Aqueous stock solutions of 1c (3 mM), Triton X-100 (8%), Tergitol (9%) and Py-NO (57 mM) were prepared in Millipore water. Samples with varying ratios of each component were prepared in vials and brought to a total volume of 1.8 ml with water.

The samples were heated to 85 °C for 30 minutes in a block heater, followed by cooling back to room temperature for 10 minutes, during which the coacervate phase settled on the bottom of the vials and turbidity disappeared. After the cooling down period 1 ml of the supernatant was withdrawn and filtered through 4.5 µm filters to HPLC vials for the reverse phase HPLC analysis.

Solvent A in the HPLC analysis was 10 mM H_3_PO_4_/KH_2_PO_4_ at pH 3, and solvent B was acetonitrile. The column used was Gemini C18, 100 × 4.6 mm with 3 µm particle size.

The gradient used in HPLC run was from 10% to 90% solvent B over 5 minutes, 90% B for 1 minute, from 90% to 10% B over 0.1 minutes, 10% B for 4 minutes. The flow rate in analysis was 1.5 ml min^−1^. The detection wavelength was UV-Vis at 280 nm, with reference at 360 nm.

The retention times of the analyte and extractants were: Py-NO, 1.6 minutes; 1c, 4.0 minutes; TX-100, 7.0 minutes. The retention time of Tergitol was not determined due to its UV inactivity. The calibration was done with 5, 10, 25, 50 and 100 ppm solutions of Py-NO before each determination. The concentration of Py-NO in the supernatant solution was determined using a calibration line. The extraction efficiency (EE_%_) is calculated by subtracting the percentage of the remaining Py-NO from 100% eqn (3).3
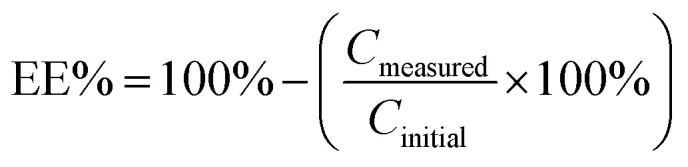


## Conclusions

Neutral water-soluble aryl-extended calix[4]pyrroles were synthesized by PEGylation, and their co-micellization with the surfactant TX-100 was assessed with DLS and cloud point temperature measurements. Longer PEG-chains including PEG-900 and PEG-550 provided miscibility with water, and CMC values below 1 mM. The effect on the cloud point temperature and micelle size indicated that calix[4]pyrroles without PEG-chains (1 and 2) soluble in organic solvents increased the micelle size more than the PEGylated derivatives, whereas the largest reduction on the cloud point temperature was induced by 1 and 1a bearing hydroxyl substituents or short PEG chains, respectively. At equimolar ratio of Triton X-100 and water-soluble 1c interactions between calix[4]pyrrole and surfactant were observed in NOESY NMR indicating that the components form mixed micelles.

Host–guest complexes of calix[4]pyrroles 1a, 2a, 1b and 1c with Py-NO and Phe-Py-NO were obtained in deuterated DMSO and water displaying diagnostic signals for specific 1 : 1 binding in the calix[4]pyrrole cavity. In DMSO, the binding affinities were modest, in the order of *K*_a_ = 10^2^ M^−1^. The high binding affinity (*K*_a_ > 10^4^ M^−1^) between calix[4]pyrrole 1c and Py-NO in water was utilized in cloud point extraction of Py-NO with a mixture of 1c and TX-100 or Tergitol. Best extraction efficiency of 92.7 ± 2.2% was obtained when using five-fold excess of the host relative to the guest alongside 2.7% Tergitol in the solution. CPE of Py-NO only succeeded with the added host because Py-NO was extracted as a 1c⊃Py–NO complex, where 1c⊃Py-NO complexes likely formed mixed micelles with non-ionic surfactants. Alternatively, the micelles of 1c with bound Py-NO aggregated with the surfactant micelles at cloud point. The results indicate that PEGylated calix[4]pyrroles can be used in conjunction with non-ionic surfactants to separate Py-NO from aqueous solution. Furthermore, the findings support the use of Tergitol as an environmentally friendly alternative to TX-100 in CPE. This opens the way for further studies of the host-assisted cloud point extraction of highly water-soluble analytes, which cannot be extracted with traditional non-ionic surfactant systems.

## Author contributions

Conceptualization K. H.; data curation J. N.; formal analysis J. N.; funding acquisition K. H.; investigation A. K., J. N., P. P. P., T. O. L.; supervision J. N., K. H., T. O. L.; visualization J. N.; writing – original draft J. N., K. H., T. O. L.; writing – review & editing J. N., K. H., T. O. L.

## Conflicts of interest

There are no conflicts to declare.

## Supplementary Material

RA-015-D5RA08163G-s001

RA-015-D5RA08163G-s002

RA-015-D5RA08163G-s003

RA-015-D5RA08163G-s004

## Data Availability

The data supporting this article have been included as part of the supplementary information (SI). Supplementary information: experimental procedures and NMR data. See DOI: https://doi.org/10.1039/d5ra08163g.
