# Supplementary figures and images for: Cloud point extraction of pyridine N-oxides using mixed micelles of PEGylated calix[4]pyrroles and non-ionic surfactants
Source: RSC Adv. 2025 Dec 15;15(58):49981–9. doi: 10.1039/d5ra08163g (PMC12703696; doi:10.1039/d5ra08163g)

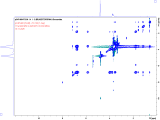

Supplement: RA-015-D5RA08163G-s003 [file RA-015-D5RA08163G-s003.zip › NOESY-1c+TX-100/14/pdata/1/thumb.png]

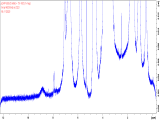

Supplement: RA-015-D5RA08163G-s003 [file RA-015-D5RA08163G-s003.zip › NOESY-1c+TX-100/4/pdata/1/thumb.png]
