# Supplementary material for: Cloud point extraction of pyridine N-oxides using mixed micelles of PEGylated calix[4]pyrroles and non-ionic surfactants
Source: RSC Adv. 2025 Dec 15;15(58):49981–9. doi: 10.1039/d5ra08163g (PMC12703696; doi:10.1039/d5ra08163g)
Supplement: RA-015-D5RA08163G-s004 [file RA-015-D5RA08163G-s004.pdf]

Supplementary Information (SI) for RSC Advances.
This journal is © The Royal Society of Chemistry 2025

Supplementary information

Contents

Instrumentation 2

Synthesized compounds and guests used in titrations and CPE 3

Characterization of synthesized compounds 4

1 4

2 5

m-PEG4-Ts 7

m-PEG550-Ts 8

1a 11

2a 12

1b 13

1c 15

Host-guest titration 18

Spectra 19

Micellar studies 28

CMC, cloud point and water solubility 28

Particle sizes and cloud point of TX-100/calix[4]pyrrole mixed micelles 29

Cloud point extraction of Py-NO with **1c** and surfactant: Examples of chromatograms 36

Control; no TX-100 or pC4P-900 36

Variable equivalents of calixpyrrole to Py-NO 37

Variable concentration of TX-100 38

Variable concentration of Tergitol 15-S-7 39

Results 40

References 42

Instrumentation

Flash chromatography was performed on a CombiFlash NextGen 300. NMR characterization and titration spectra were recorded using a Bruker Avance III 500 MHz spectrometer with TXI prodigy probe head. NOESY was performed on a Bruker Avance III 400 MHz spectrometer with BBFO probe. Spectra were processed and analyzed using Topspin 4.0.8 and figures were prepared using MestreNova. IR spectra were measured on a Bruker Alpha FT-IR. MS were acquired with an Agilent 630 UHPLC-QTOF spectrometer. Dynamic light scattering data were recorded using a Malvern Panalytical Zetasizer Ultra. pH values were measured using a Mettler Toledo FiveEasy F20. Micelle samples were heated with a Labnet International Accublock Mini Digital Dry Bath. HPLC was run on an HP Agilent series 1100 HPLC system with a DAD detector.

Synthesized compounds and guests used in titrations and CPE

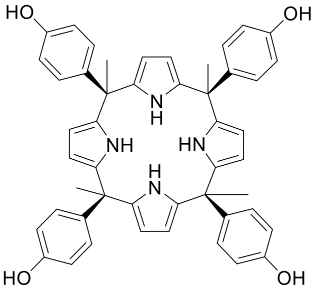

1

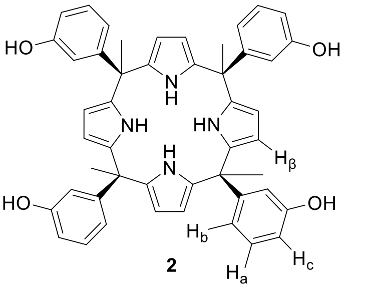

2

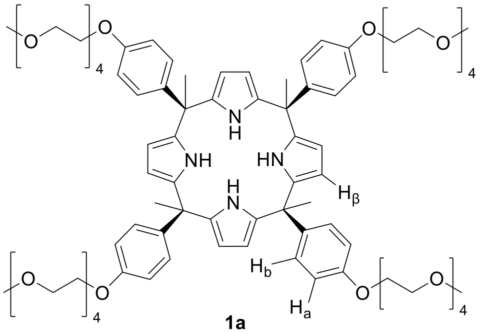

1a

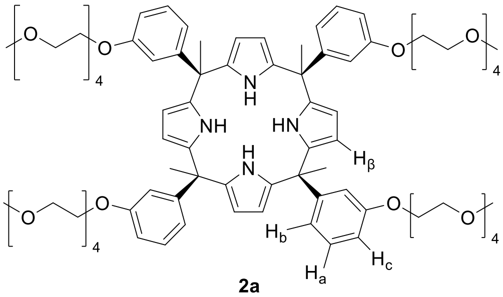

2a

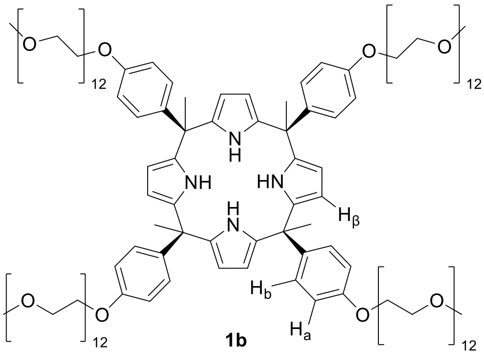

1b

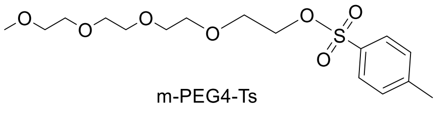

m-PEG4-Ts

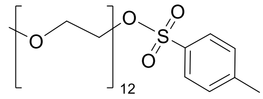

m-PEG550-Ts

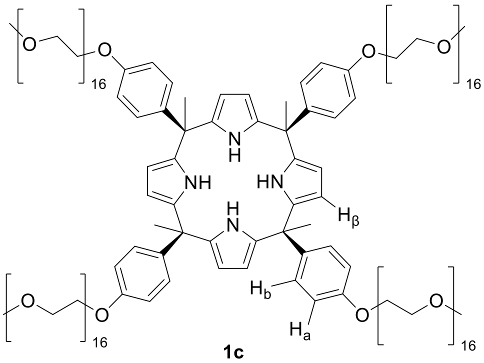

1c

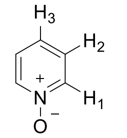

pyridine *N*-oxide, Py-NO

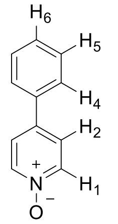

p-phenylpyridine *N*-oxide, Phe-Py-NO

Figure S1. Synthesized compounds **1**, **2**, **1a-c** and **2a**, alongside guests pyridine *N*-oxide (Py-NO) and *p*-phenylpyridine *N*-oxide (Phe-Py-NO). Proton assignations for titration data are shown as subscript.

Characterization of synthesized compounds

1[1–3]

^{1}H NMR (DMSO-d$_6$, 500 MHz) δ (ppm) 1.71 (s, 12H, CH$_3$), 5.93 (d, J = 2.6 Hz, 8H, H$_\beta$), 6.62 (d, J = 8.4 Hz, 8H, Ph), 6.68 (d, J = 8.4 Hz, 8H, Ph), 9.26 (s, 4H, OH), 9.43 (s, 4H, NH).

^{13}C NMR (DMSO-d$_6$, 126 MHz) δ (ppm) 31.6 (C$_6$), 43.4 (C$_5$), 104.2 (C$_8$), 114.9 (C$_1$), 127.9 (C$_2$), 137.5 (C$_3$), 140.5 (C$_4$), 155.9 (C$_7$).

HRMS (ESI-Q-TOF) *m/z*: calcd. for $C_{48}H_{45}N_4O_4^+$ 741.3435; found 741.3419.

IR (ν_{max} cm^{-1}): 3363 (N-H stretch), 3155 (O-H stretch), 2933 (C-H stretch), 1613 (C=C stretch), 766 (C-H aromatic bend).

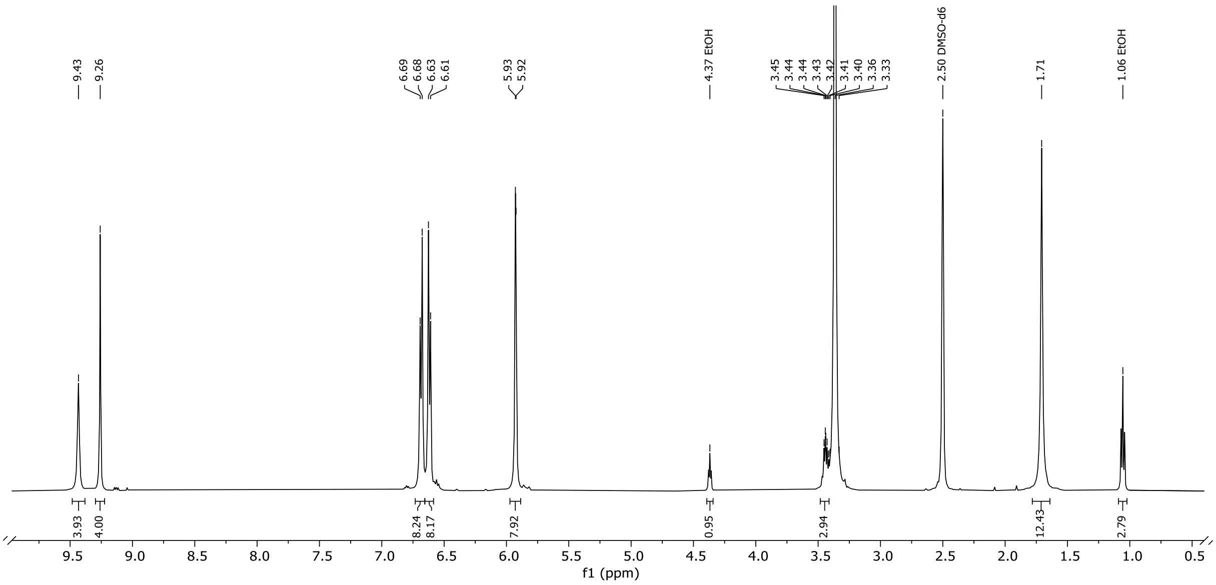

Figure S2. ^{1}H NMR of **1**.

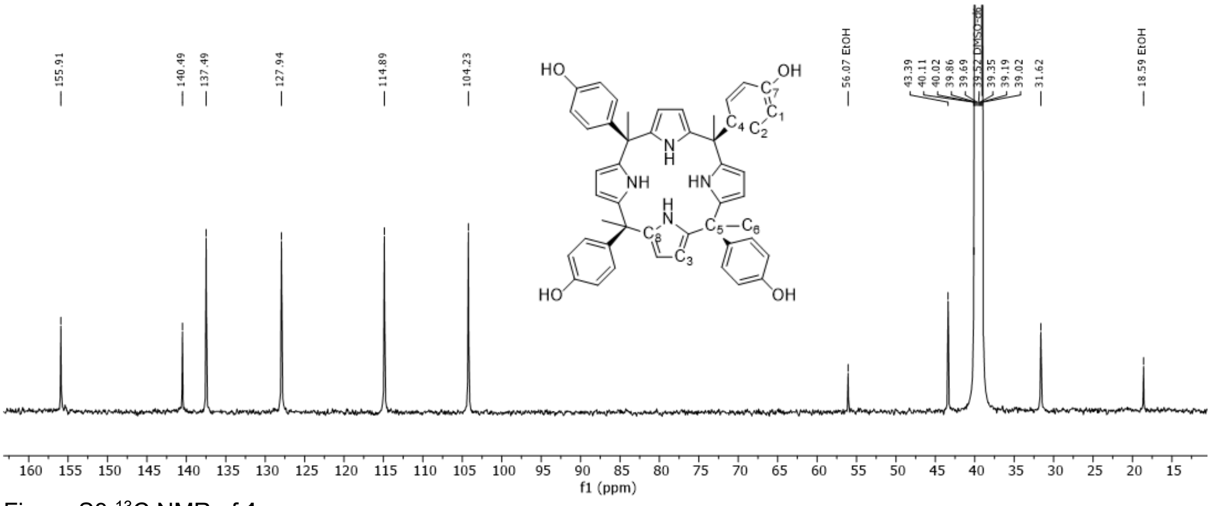

Figure S3 ^{13}C NMR of **1**.

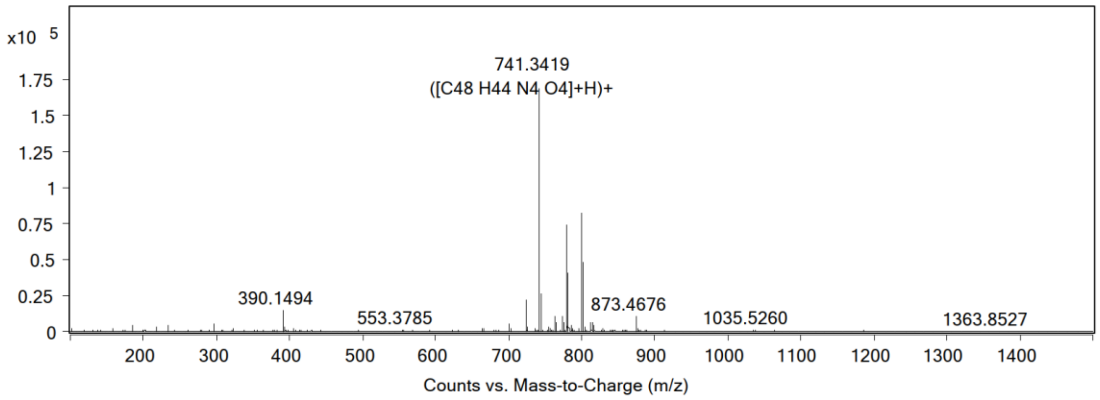

Figure S4. HRMS (ESI-Q-TOF) spectrum of **1**, proton adduct at positive polarization.

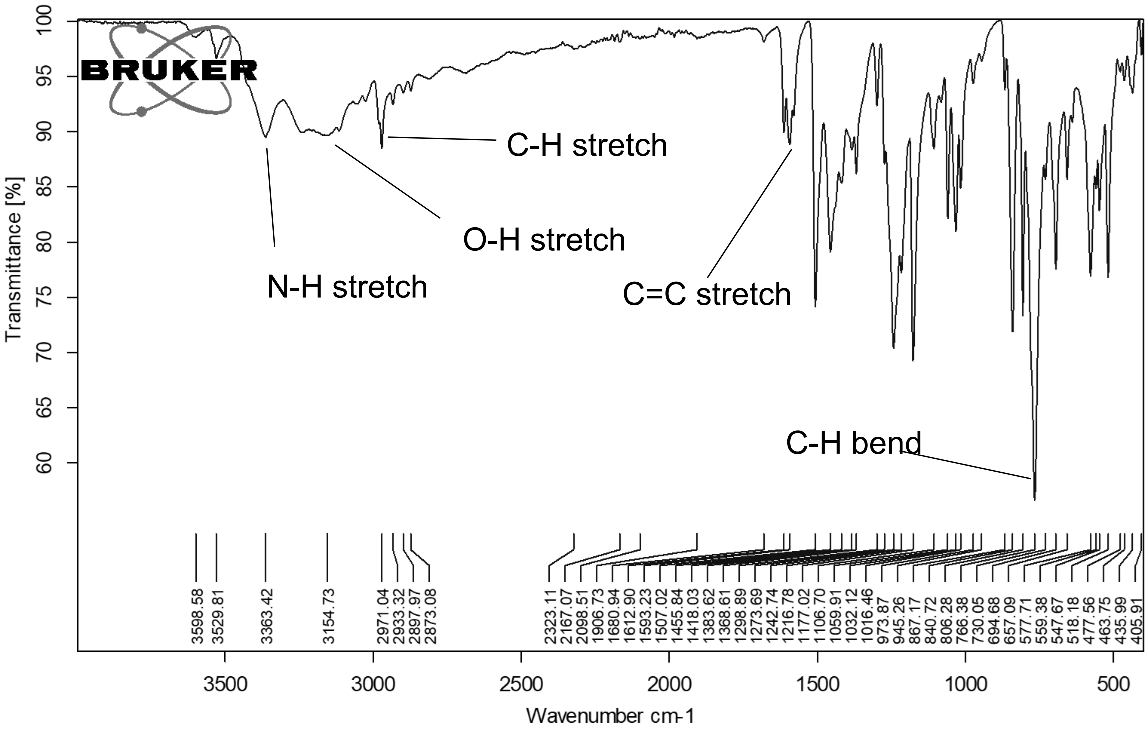

Figure S5. IR spectrum of **1**.

2[4]

^{1}H NMR (500 MHz DMSO-d$_6$) *δ* (ppm) 1.76 (s, 12H, CH_3), 5.97 (d, *J* = 2.5 Hz, 8H, $H_β$), 6.38-6.41 (m, 8H, Phe), 6.45 (dd, *J* = 8.0, 1.4 Hz, 4H, Phe), 6.98 (t, *J* = 8.0 Hz, 4H, Phe), 9.09 (s, 4H, OH), 9.47 (s, 4H, NH).

^{13}C (126 MHz DMSO-d$_6$) *δ* (ppm) 31.3 (C_8), 44.1 (C_7), 104.2 (C_{10}), 113.2 (C_3), 114.0 (C_1), 117.9 (C_5), 129.1 (C_4), 137.3 (C_9), 151.8 (C_6), 157.4 (C_2).

HRMS (ESI-Q-TOF), *m/z*: $[M+H]^+$ calcd. for $C_{48}H_{45}N_4O_4^+$ 741.3435; found 741.3414.

IR ($ν_{max}$, cm^{-1}): 3406 (N-H stretch), 3105 (O-H stretch), 1579 (C=C stretch), 750 (aromatic C-H bend).

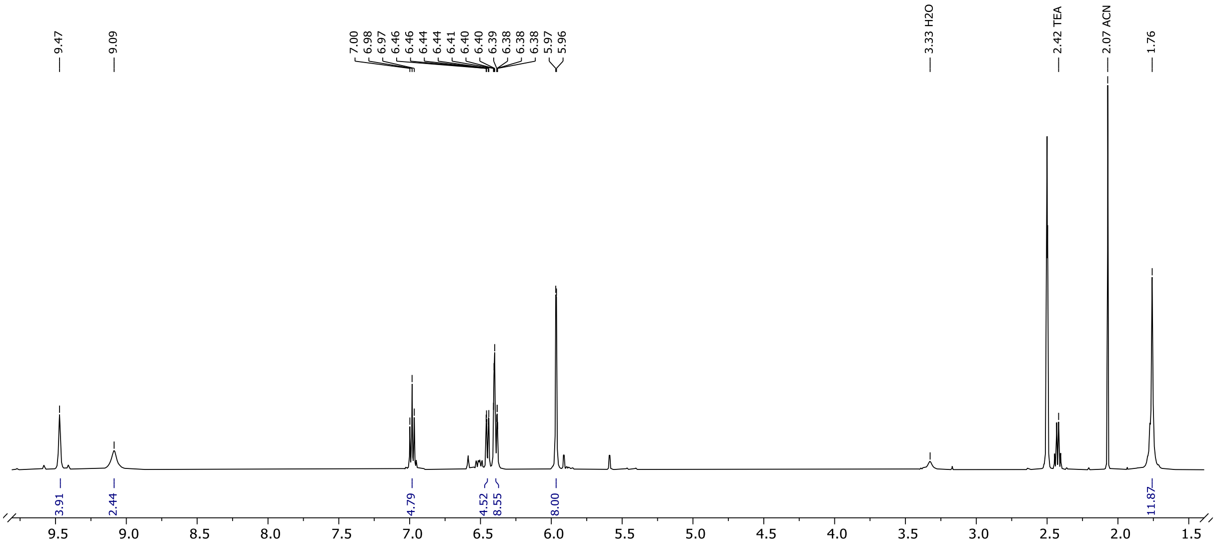

Figure S6. ^{1}H NMR of **2**. 500 MHz, DMSO-d6.

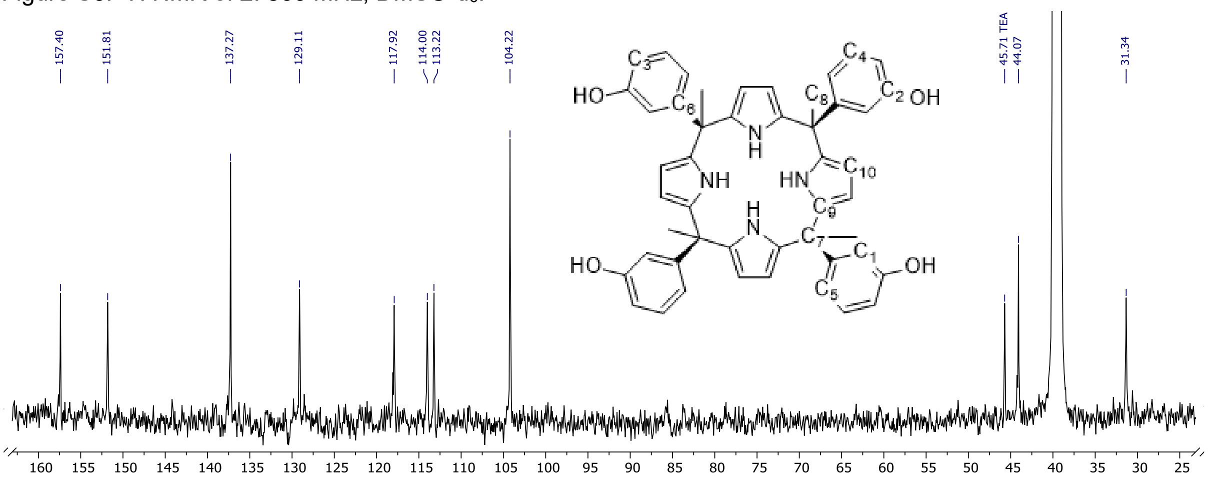

Figure S7. ^{13}C NMR of **2**. 126 MHz, DMSO-d6.

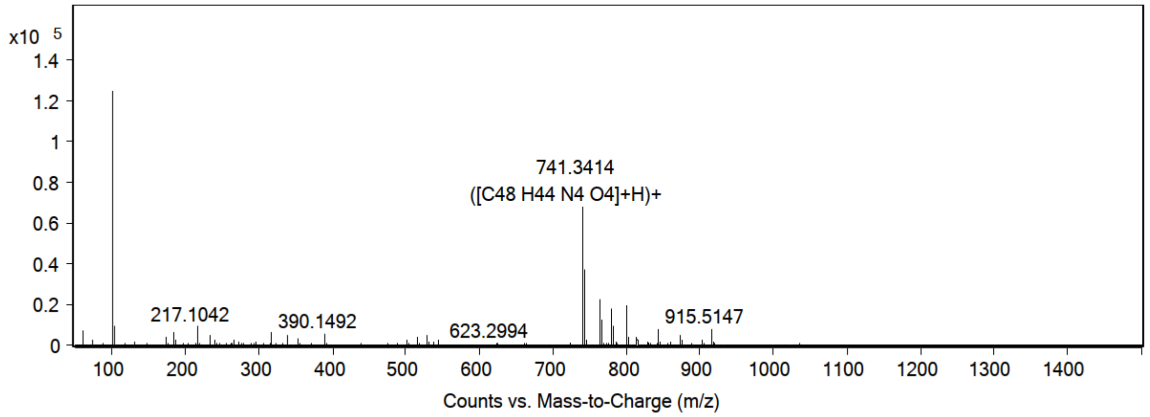

Figure S8. HRMS (ESI-Q-TOF) of **2**, proton adduct at positive polarization.

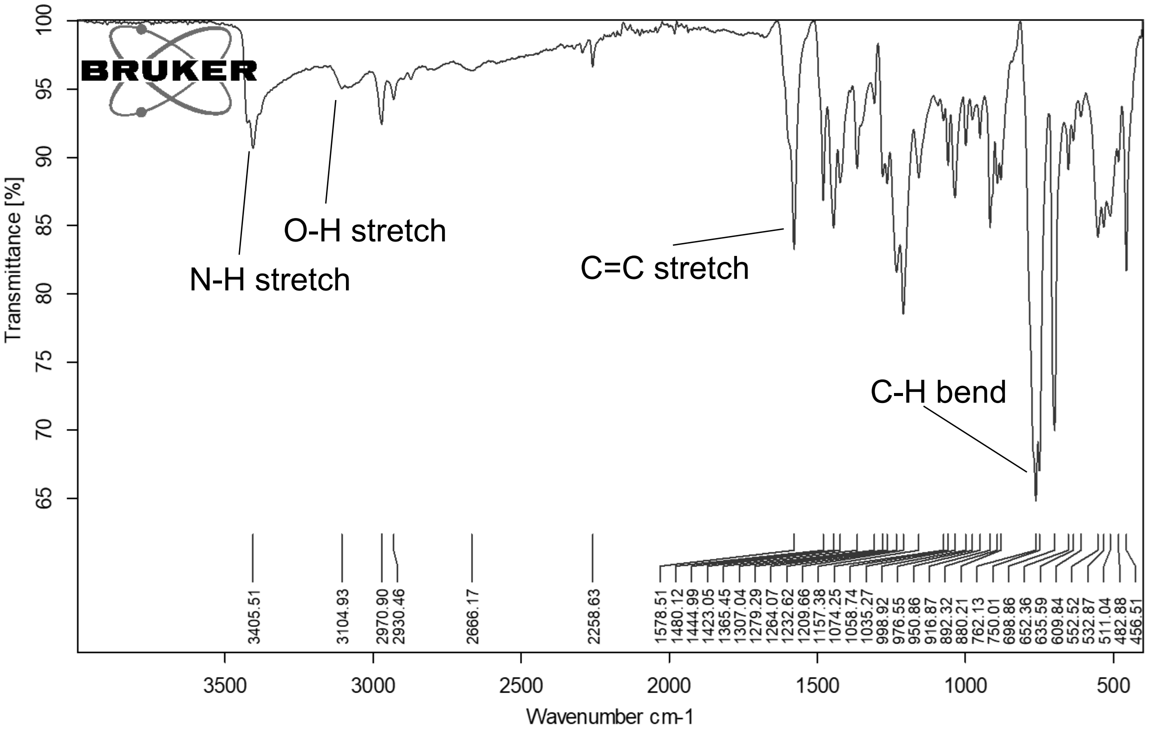

Figure S9. IR spectrum of **2**.

m-PEG4-Ts[5]

^{1}H NMR (MeOD, 500 MHz) *δ* (ppm) 2.45 (s, 3H, CH_3), 3.35 (s, 3H, OCH_3), 3.52-3.56 (m, 6H, PEG), 3.59-3.62 (m, 6H, PEG), 3.65 (m, 2H, PEG), 4.14 (m, 2H, PEG), 7.44 (d, *J* = 8.0, 2H, Phe), 7.80 (d, *J* = 8.0 Hz, 2H, Phe).

^{13}C NMR (MeOD, 126 MHz) *δ* (ppm) 21.6 (C_4), 59.1 (C_5), 69.7 (PEG), 70.9 (PEG), 71.3 (PEG), 71.5 (PEG), 72.9 (PEG), 129.0 (C_1), 131.1 (C_2), 134.4 (C_3), 146.4 (C_6).

HRMS (ESI-Q-TOF) *m/z*: $[M+Na]^+$ calcd. for $C_{16}H_{26}O_7SNa^+$ 385.12915; found 385.1302, $[M+K]^+$ calcd. for $C_{16}H_{26}O_7SNa^+$ 401.10308; found 401.1029.

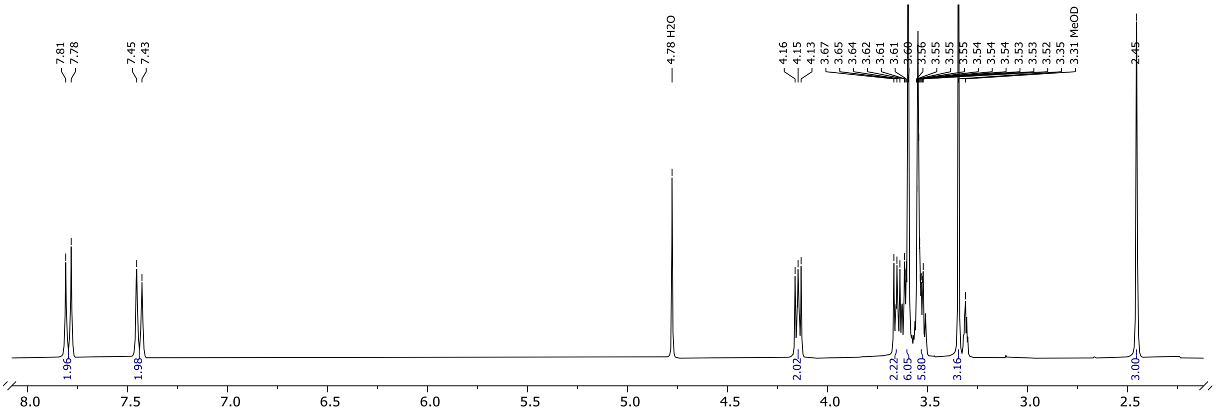

Figure S10. ^{1}H NMR of m-PEG4-Ts. 500 MHz, MeOD.

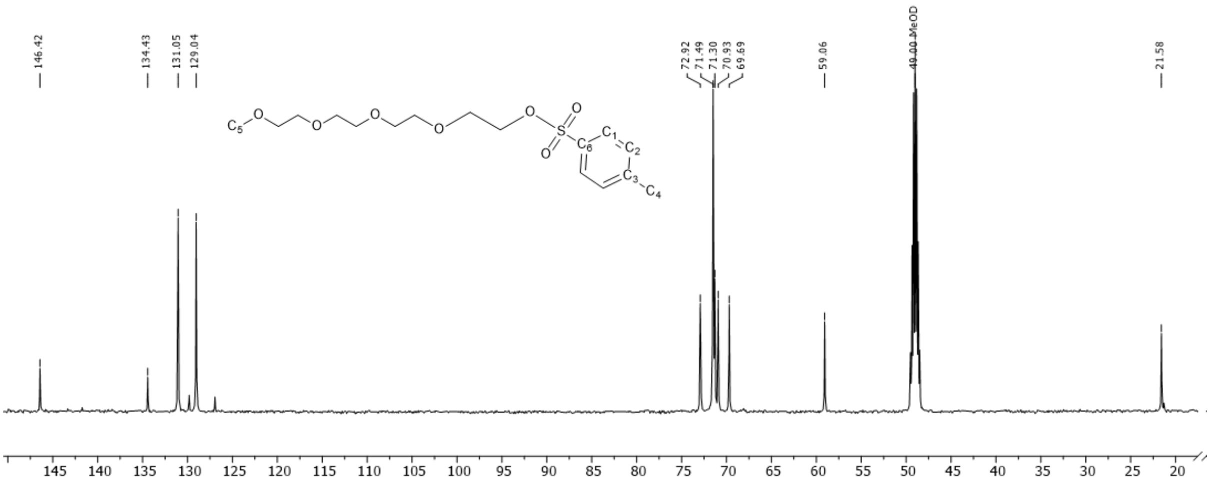

Figure S11. ^{13}C NMR of m-PEG4-Ts. 126 MHz, MeOD.

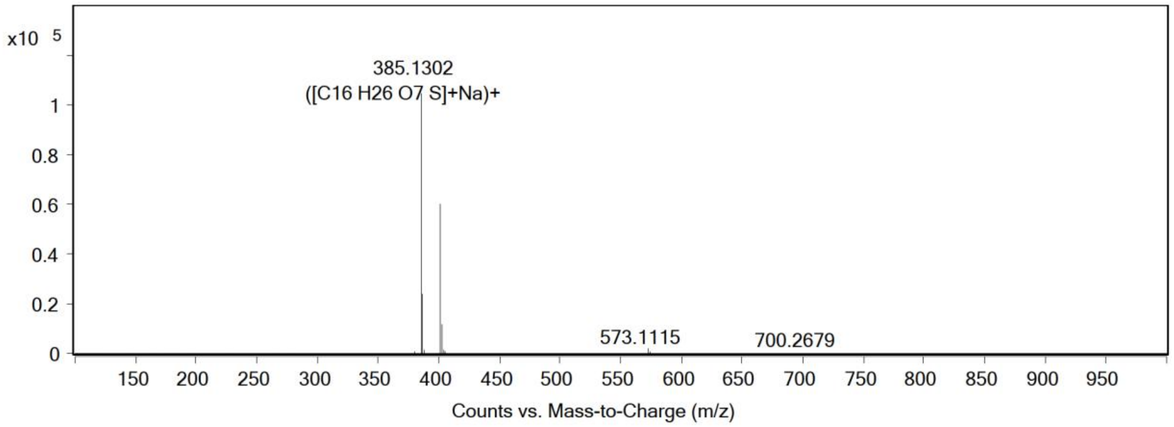

Figure S12. HRMS (ESI-Q-TOF) of m-PEG4-Ts, sodium adduct at positive polarization.

m-PEG550-Ts[6]

^{1}H NMR (DMSO-d6, 500 MHz) *δ* (ppm): 2.42 (s, 3H, CH_3), 3.24 (s, 3H, OCH_3), 3.42–3.44 (m, 6H, PEG), 3.48–3.58 (m, 32H, PEG), 3.57 (t, *J* = 5.0 Hz, 2H, PEG), 4.11 (t, *J* = 5.0 Hz, 2H, PEG), 7.48 (d, *J* = 8.0 Hz, 2H, Phe), 7.78 (d, *J* = 8.0 Hz, 2H, Phe).

^{13}C NMR (DMSO-d6, 126 MHz) *δ* (ppm) 21.1 (C_4), 58.1 (C_5), 67.9 (PEG), 70.0 (PEG), 71.3 (PEG), 72.0 (PEG), 127.6 (C_1), 130.1 (C_2), 132.4 (C_3), 144.9 (C_6).

IR (cm^{-1}): 1094 (C–O–C stretch), 1351 (S=O stretch), 1452, 1598 (C–C stretch), 2866 (C–H stretch).

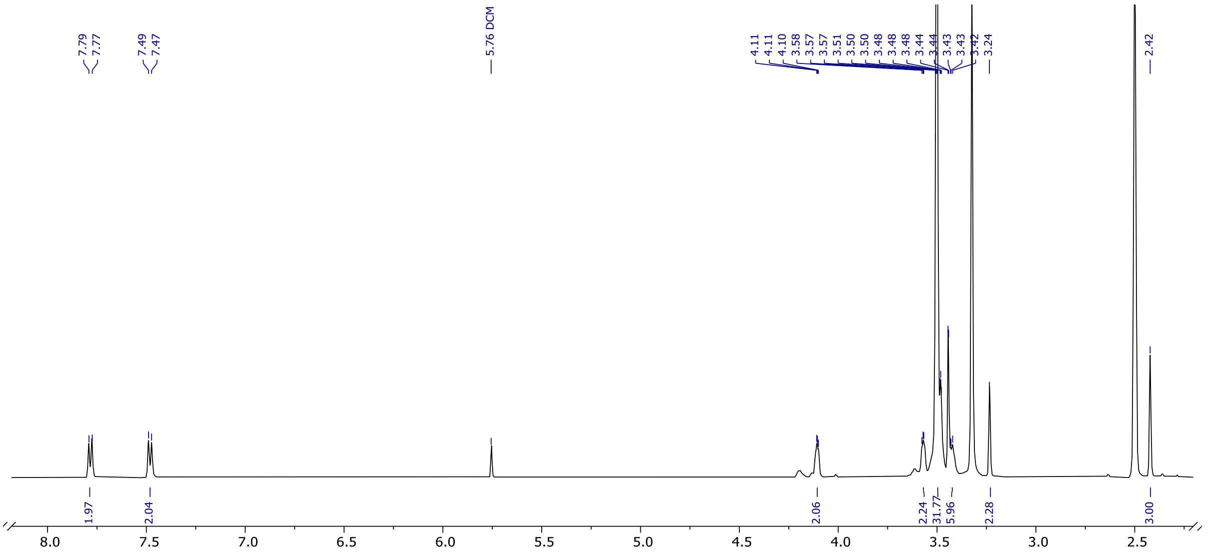

Figure S13. ^{1}H NMR of m-PEG550-Ts. 500 MHz, DMSO-d_6.

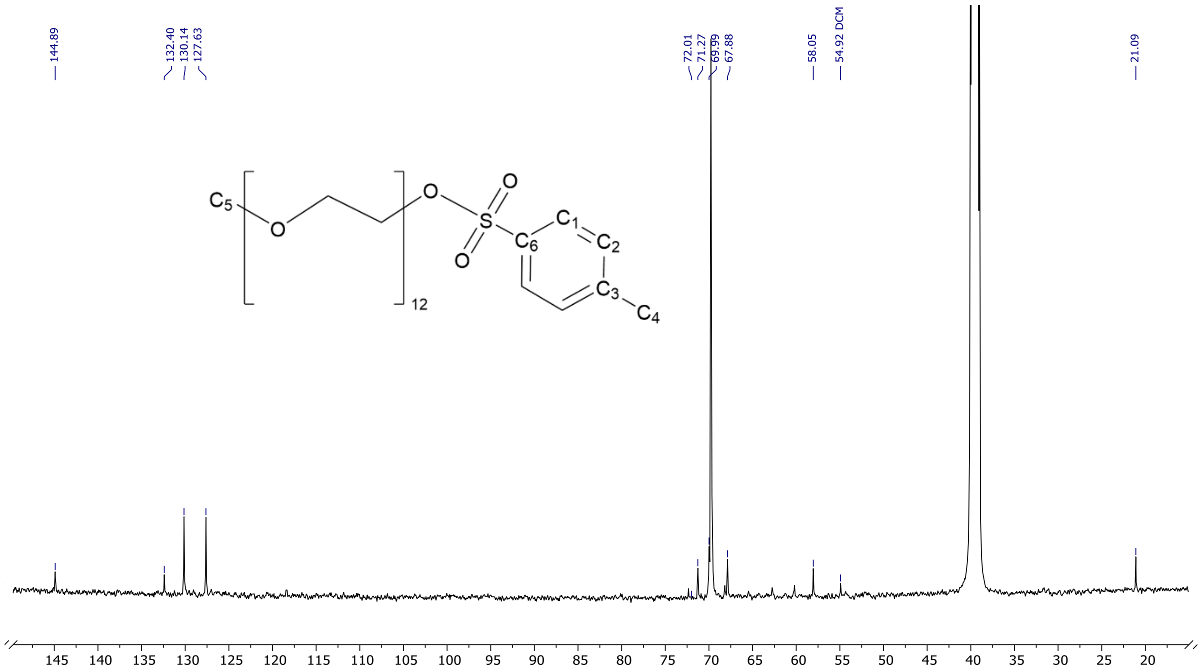

Figure S14. ^{13}C NMR of m-PEG550-Ts. 126 MHz, DMSO-d_6.

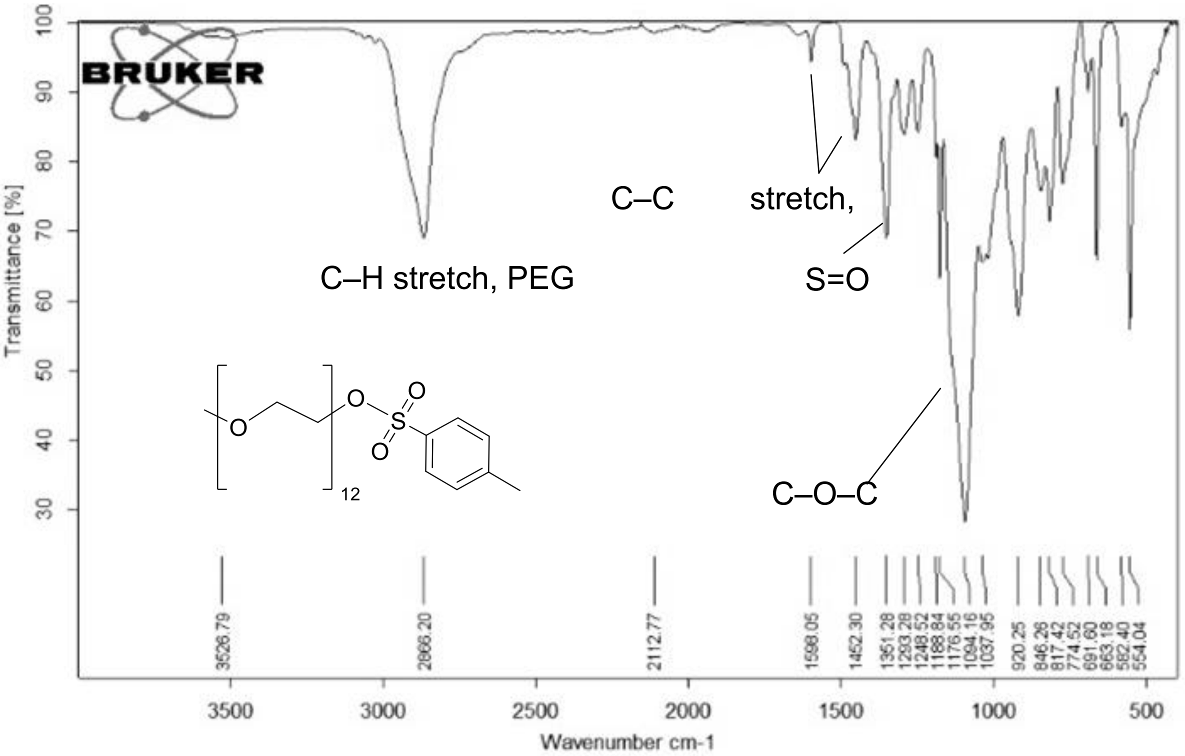

Figure S15. IR spectrum of m-PEG550-Ts.

1a

^{1}H NMR (500 MHz DMSO-d$_6$) δ 1.74 (s, 12H, CH_3), 3.23 (s, 12H, OCH_3), 3.41-3.43 (m, 12H, PEG), 3.49-3.55 (m, 48H, PEG), 3.67 (t, J = 4.8 Hz, 8H, PEG), 3.99 (t, J = 4.8 Hz, 8H, PEG), 5.93 (s, 8H, H$_\beta$), 6.78 (d, J = 8.7 Hz, 8H, Phe), 6.90 (d, J = 8.9 Hz, 8H, Phe), 9.42 (s, 4H, OH).

^{13}C (126 MHz DMSO-d$_6$) δ 31.3 (C$_2$), 43.4 (C$_1$), 58.1 (C$_8$), 67.2 (PEG), 68.8 (PEG), 69.6 (PEG), 69.8 (PEG), 69.9 (PEG), 71.3 (PEG), 104.5 (C$_9$), 114.3 (C$_5$), 127.9 (C$_4$), 137.4 (C$_7$), 142.2 (C$_3$), 157.2 (C$_6$).

HRMS (ESI-Q-TOF) *m/z*: calcd. [M+Cl]$^+$ for $C_{84}H_{116}N_4O_{20}Cl^-$ 1535.78714; found 1535.7887.

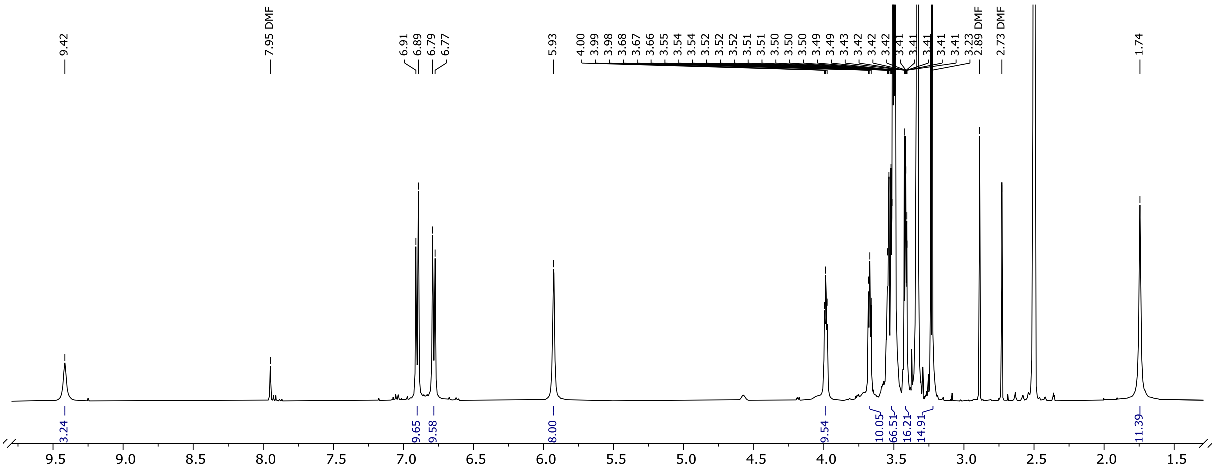

Figure S16. ^{1}H NMR of **1a**. 500 MHz, DMSO-d$_6$.

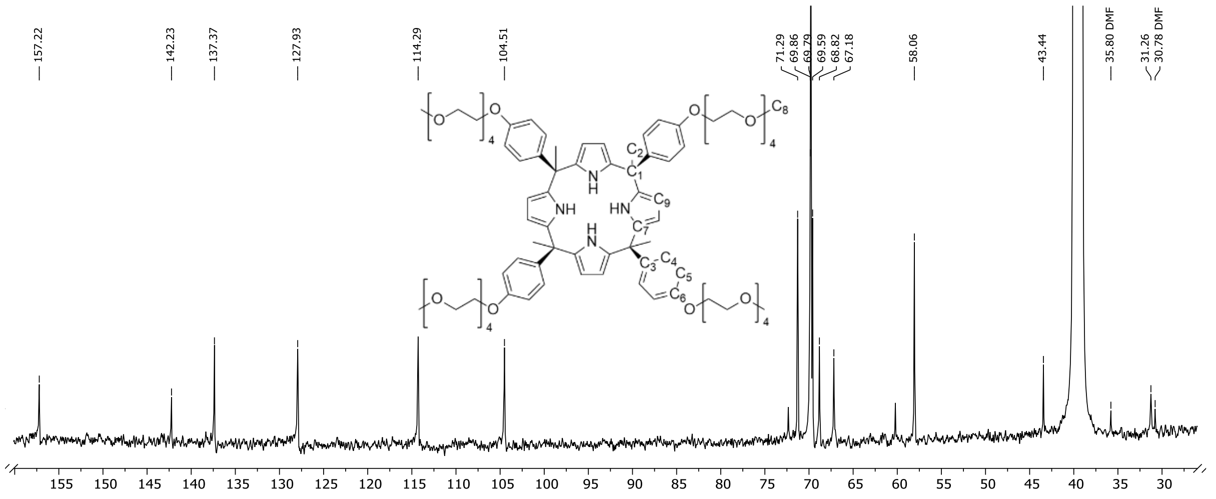

Figure S17. ^{13}C NMR of **1a**. 126 MHz, DMSO-d$_6$.

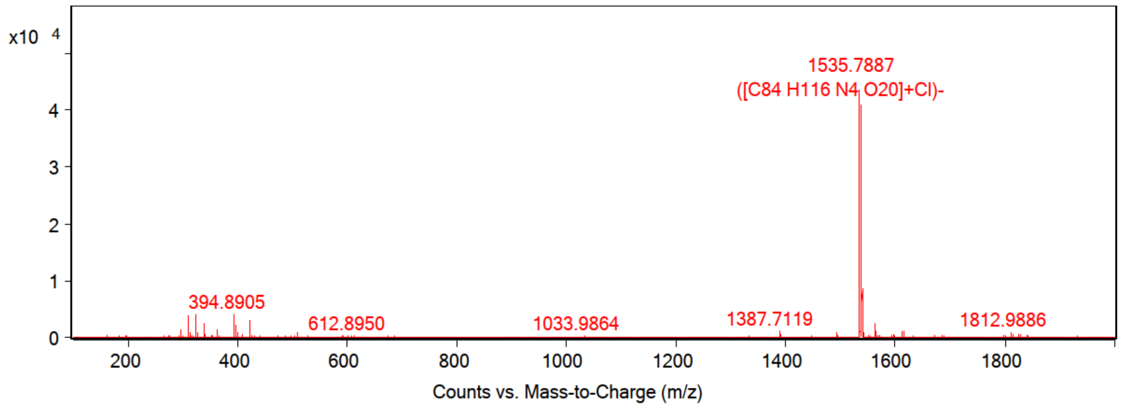

Figure S18. HRMS (ESI-Q-TOF) of **1a**, chloride adduct at negative polarization.

2a

^{1}H NMR (500 MHz DMSO-d6) δ (ppm) 1.79 (s, 12H, CH_3), 3.22 (s, 12H, OCH_3), 3.39-3.44 (m, 18H, PEG), 3.46-3.52 (m, 52H, PEG), 3.53-3.55 (m, 8H, PEG), 3.58-3.60 (m, 8H, PEG), 3.74 (t, J = 5.0, 8H, PEG), 3.98 (t, J = 5.0, 8H, PEG), 5.98 (s, 8H, H_β), 6.43-6.47 (m, 8H, Phe), 6.68 (dd, ^3J = 8.2 Hz, 2.2 Hz , 4H, Phe), 7.18 (t, J = 8.0 Hz, 4H, Phe), 9.46 (s, 4H, NH).

^{13}C NMR (DMSO- d6, 126 MHz) δ (ppm): 31.1 (C_8), 44.1 (C_7), 58.1 (C_{11}), 66.9 (PEG), 69.0 (PEG), 69.6 (PEG), 69.8 (PEG), 70.0 (PEG), 71.3 (PEG), 72.4 (PEG), 104.7 (C_{10}), 111.4 (C_3), 113.9 (C_1), 119.1 (C_5), 129.3 (C_4), 137.0 (C_9), 151.9 (C_6), 158.6 (C_2).

HRMS (ESI-Q-TOF) m/z: $[M+H]^+$ calcd. for $C_{84}H_{117}N_4O_{20}^+$ 1501.82612; found 1501.8216, $[M+K]^+$ calcd. for $C_{84}H_{116}N_4O_{20}K^+$ 1539.78145; found 1539.7795.

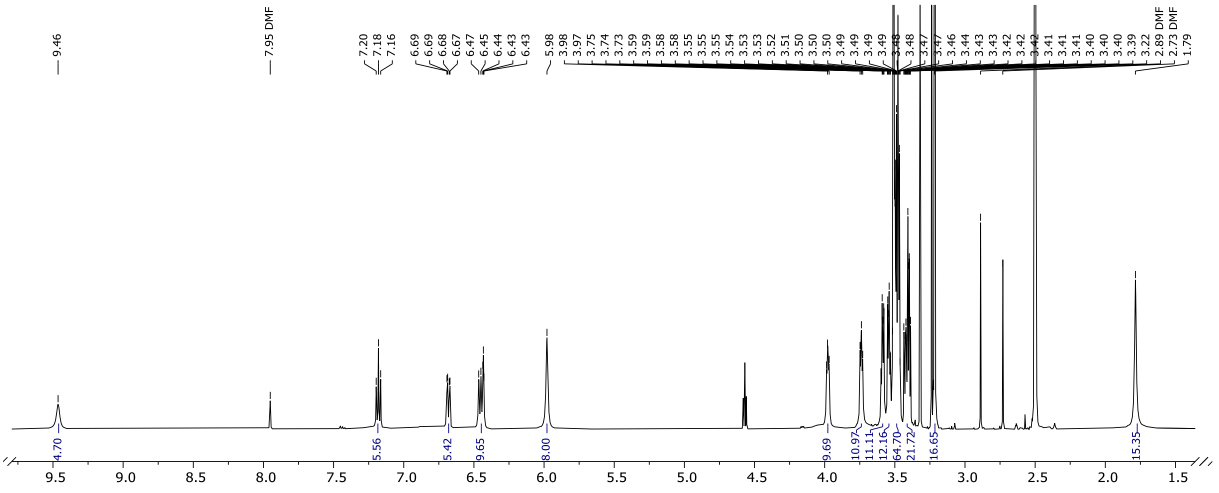

Figure S19. ^{1}H NMR of **2a**. 500 MHz, DMSO-d_6.

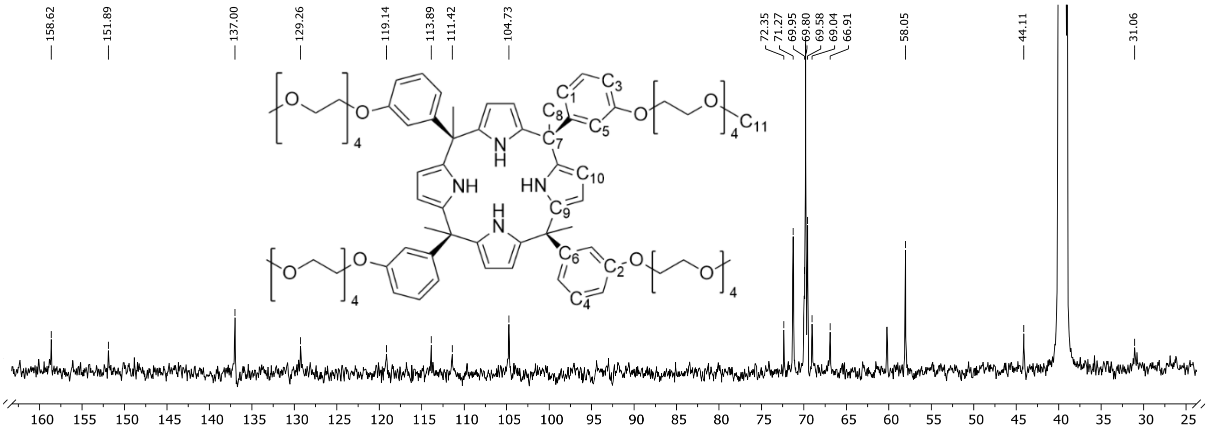

Figure S20. ^{13}C NMR of **2a**. 500 MHz, DMSO-d_6.

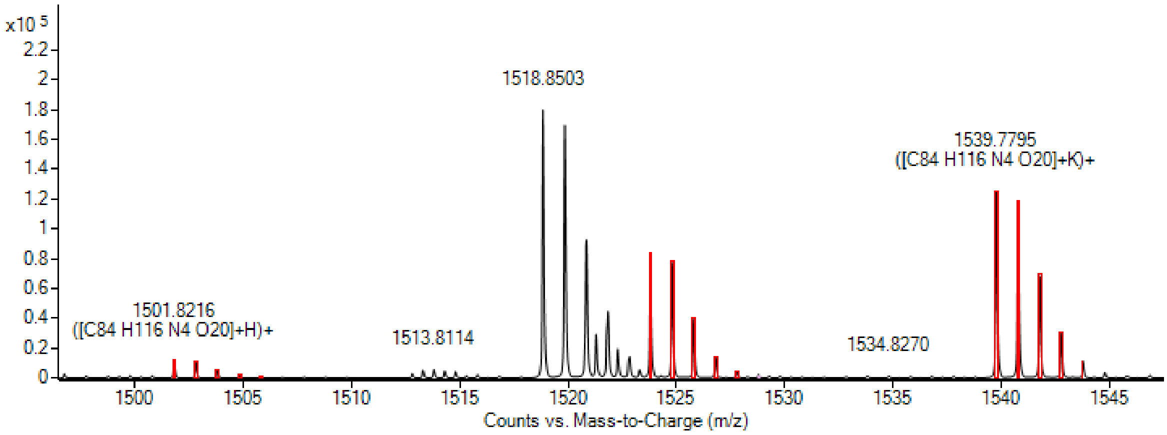

Figure S21. HRMS (ESI-Q-TOF) of **2a**, potassium and proton adducts at positive polarization.

1b

^{1}H NMR (DMSO- d_6, 500 MHz) δ (ppm): 1.74 (s, 12H, CH_3), 3.23 (s, 12H, OCH_3), 3.42 (t, J = 4.8, 12H, PEG), 3.48–3.54 (m, 250H, PEG), 3.67 (t, J = 8.5 Hz, 8H, PEG), 3.99 (t, J = 8.5 Hz, 8H, PEG), 5.92 (s, 8H, H_β), 6.78 (d, J = 8.7 Hz, 8H, Phe), 6.90 (d, J = 8.8 Hz, 8H, Phe), 9.42 (s, 4H, NH).

^{13}C NMR (DMSO- d_6, 126 MHz) δ (ppm): 31.2 (C_2), 43.4 (C_1), 58.1 (C_8), 68.8 (PEG), 69.6 (PEG), 69.8 (PEG), 71.3 (PEG), 104.5 (C_9), 114.3 (C_5), 127.9 (C_4), 137.4 (C_7), 142.2 (C_3), 157.2 (C_6).

IR (cm^{-1}): 1097 (C–O–C stretch), 1248 (C–N stretch), 1606 (N–H bend), 2867 (C–H stretch, PEG), 3359 (N–H stretch).

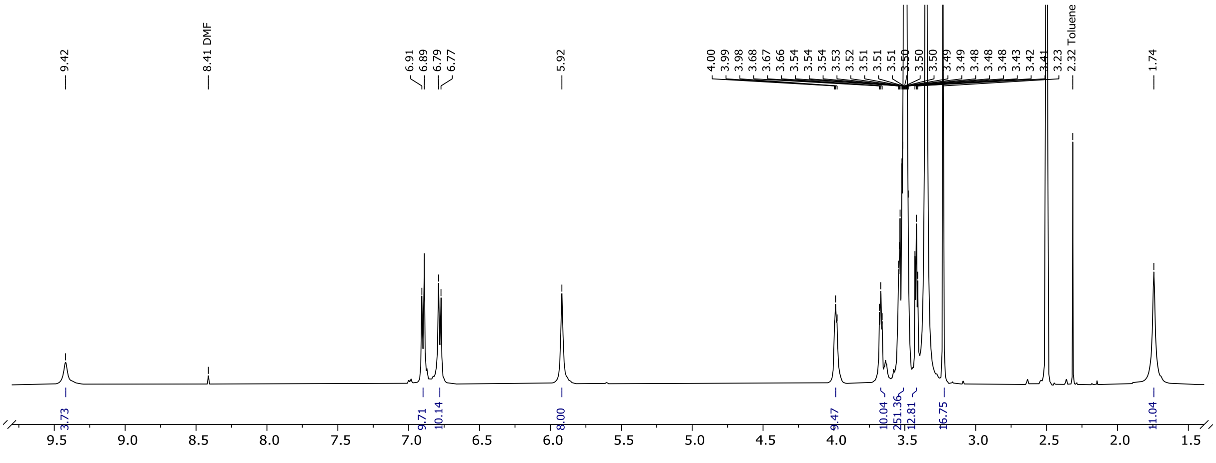

Figure S22. ^{1}H NMR of **1b**. 500 MHz, DMSO-d6.

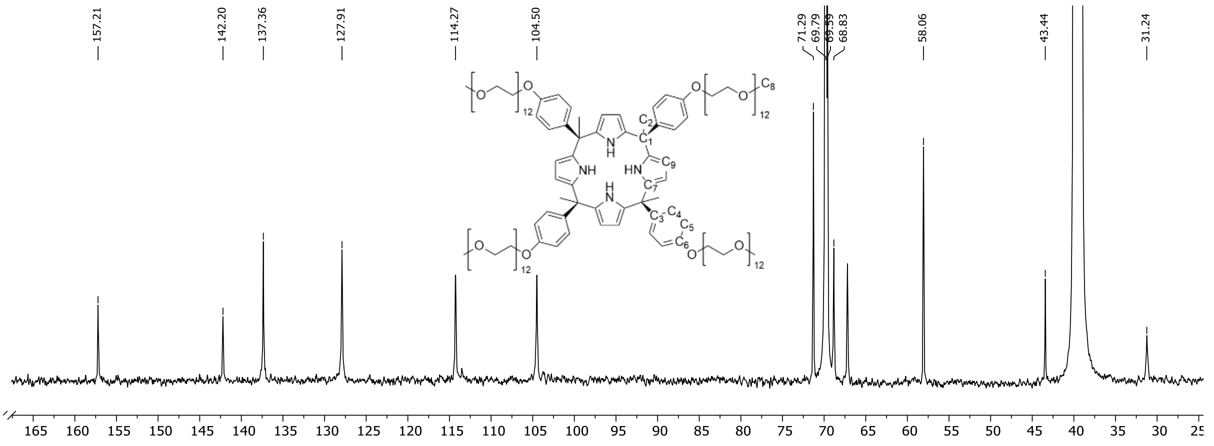

Figure S23. ^{13}C NMR of **1b**. 500 MHz, DMSO-d6.

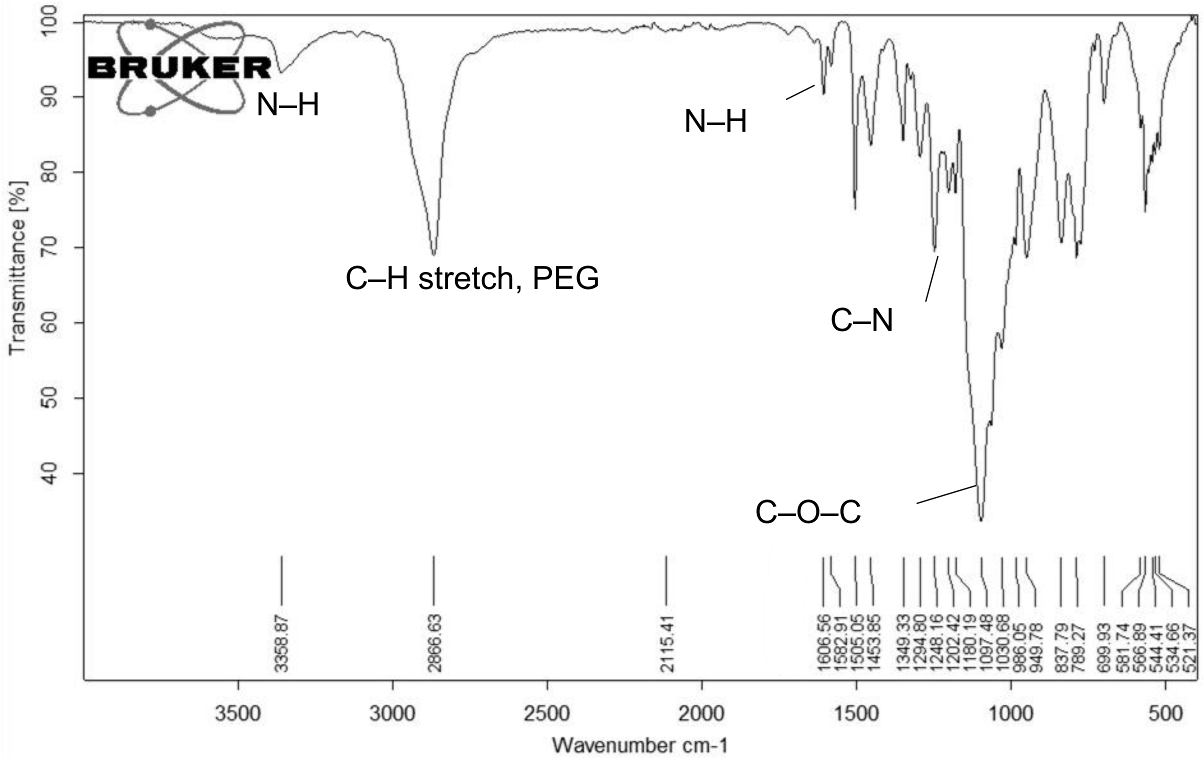

Figure S24. IR spectrum of **1b**.

1c

^{1}H NMR (500 MHz DMSO-d_6) δ 1.75 (s, 12H, CH_3), 3.24 (s, 12H, OCH_3), 3.42 (t, J = 4.5, 8H, PEG), 3.41-3.43 (m, 12H, PEG), 3.50-3.56 (m, 370H, PEG), 3.67 (t, J = 4.5, 8H, PEG), 3.99 (t, J = 4.5, 8H, PEG), 5.92 (s, 8H, H_β), 6.79 (d, J = 8.5, 8H, Phe), 6.90 (d, J = 8.5, 8H, Phe), 9.42 (s, 4H, NH).

^{13}C (126 MHz DMSO-d_6) δ 31.2 (C_2), 43.4 (C_1), 58.0 (C_8), 67.2 (PEG), 68.8 (PEG), 69.6 (PEG), 69.8 (PEG), 71.3 (PEG), 104.5 (C_9), 114.3 (C_5), 127.9 (C_4), 137.3 (C_7), 142.2 (C_3), 157.2 (C_6).

IR (cm^{-1}): 1086 (C–O–C stretch), 1257 (C–N stretch), 1606 (N–H bend), 2961 (C–H stretch, PEG), 3402 (N–H stretch).

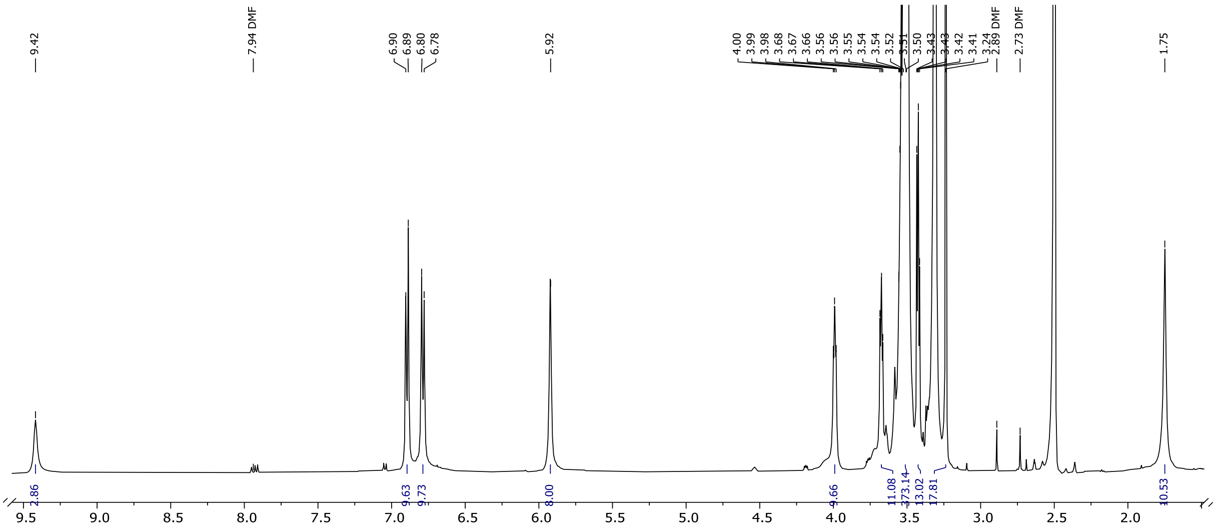

Figure S25. ^{1}H NMR of **1c**. 500 MHz, DMSO-d_6.

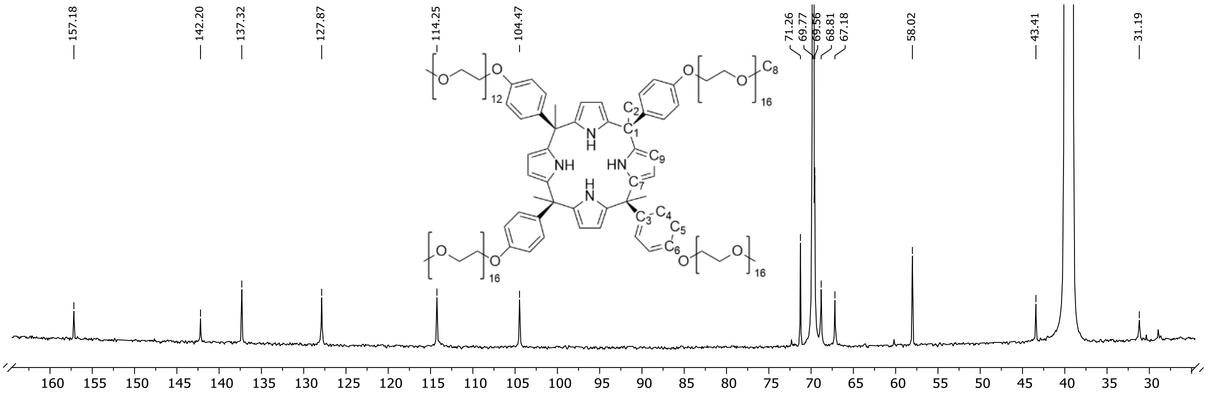

Figure S26. ^{13}C NMR of **1c**. 500 MHz, DMSO-d_6.

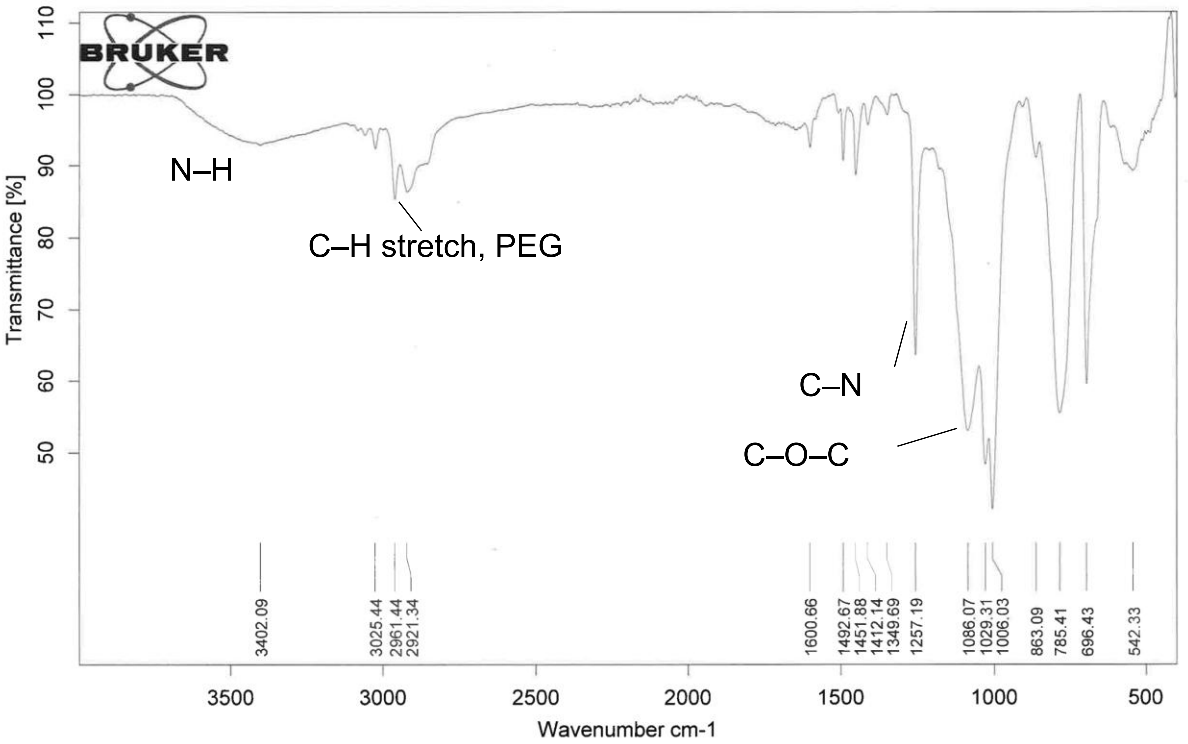

Figure S27. IR spectrum of **1c**.

Host-guest titration

First trial (Table S1) shows the result from the NMR titrations. In addition, for each host-guest pair, one sample with 1:1 ratio of calix[4]pyrrole and guest was made (trial 2). The average binding constant and standard deviation was calculated from trials 1 and 2 (Table S1).

Table S1. Binding constants (M^{-1}) between two 1:1 samples of calix[4]pyrroles **1a**, **1b** and **2a** and guests Py-NO and Phe-Py-NO.

K / M^{-1}	**1a** + PyNO	**1a** + Phe-Py-NO	**2a** + Py-NO	**1b** + Py-NO	**1b** + Phe-Py-NO
Trial 1	107.240217	182.35842	22.71238972	96.75831402	119.1157381
Trial 2	47.5456379	119.23863	3.716610142	35.60493177	122.1787721
Average binding constant / M^{-1}	77.39	150.80	13.21	66.18	120.65
Standard deviation	42.21	44.63	13.43	43.24	2.167

Spectra

Titration of Py-NO with **1a** (4.4 mM, DMSO-d_6).

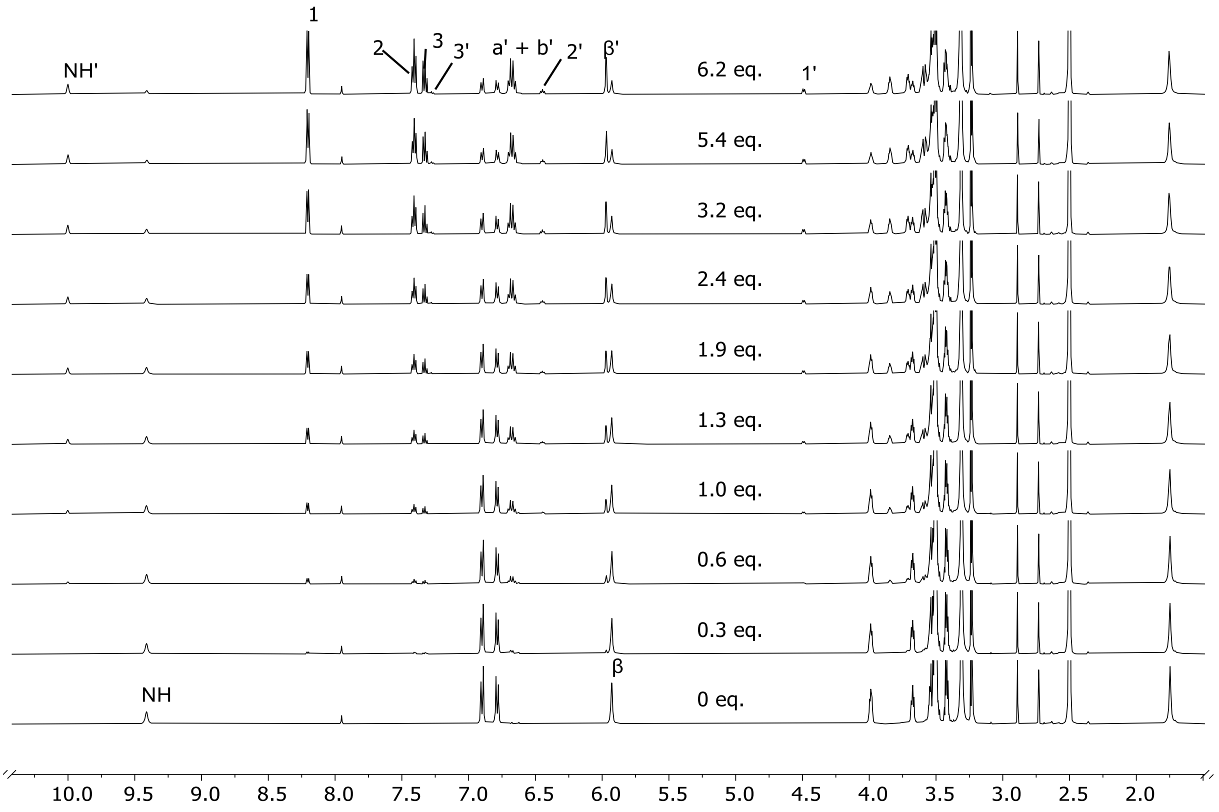

Figure S28. Titration of Py-NO with **1a** (4.4 mM, DMSO-d_6).

Table S2. Integrals of titration between **1a** and Py-NO.

Equivalents of guest	Integral of free NH / 9.41 ppm	Integral of bound NH / 10.00 ppm
0	3.9542	0
0.3	3.3751	0.02993
0.6	3.177	0.5502
1.0	2.8239	0.9527
1.3	2.7268	1.2163
1.9	2.2994	1.2904
2.4	2.0829	1.8213
3.2	1.7659	2.0184
5.4	1.7197	2.4588
6.2	1.5134	2.434

Titration of Phe-Py-NO with **1a** (5.2 mM, DMSO-d6).

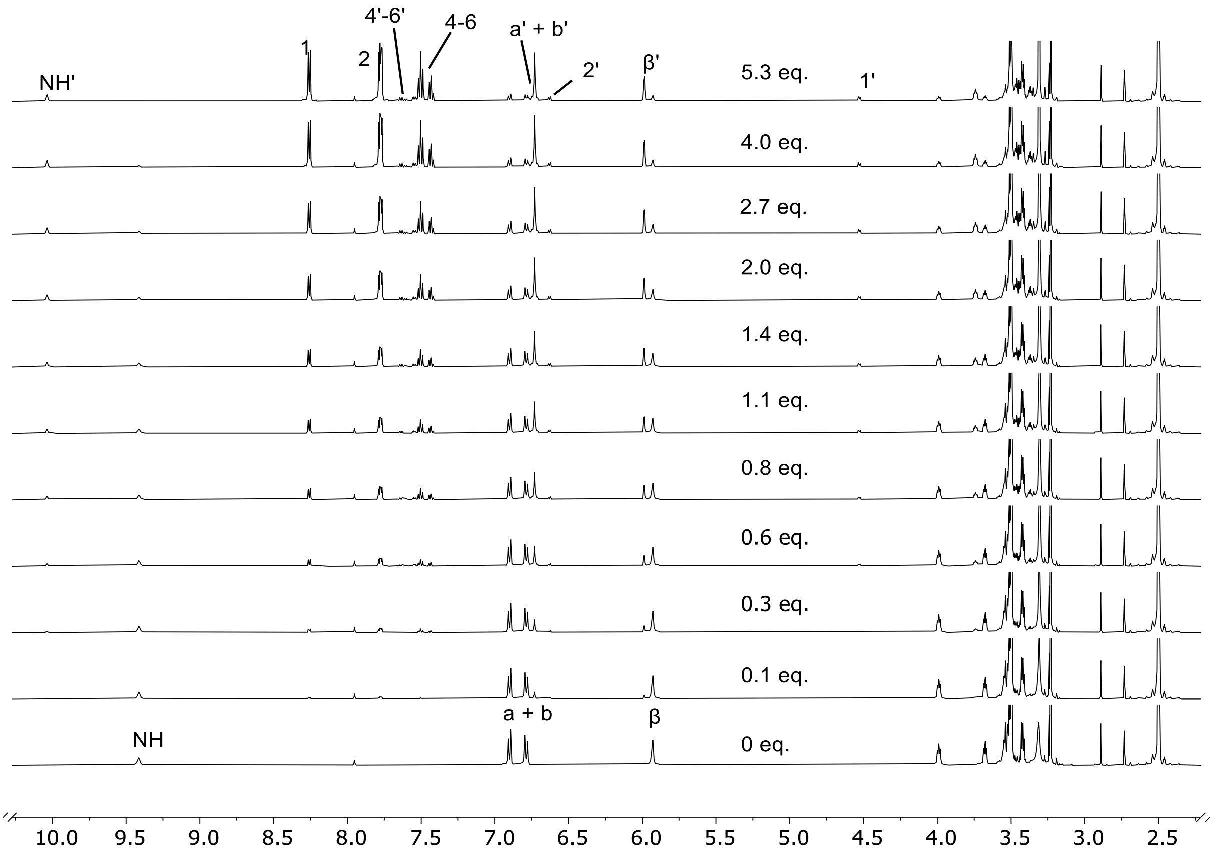

Figure S29. Titration of Phe-Py-NO with **1a** (5.2 mM, DMSO-d6).

Table S3. Integrals of titration between **1a** and Phe-Py-NO.

Equivalents of guest	Integral of free NH / 9.41 ppm	Integral of bound NH / 10.00 ppm
0	3.9872	0
0.1	3.7557	0.3728
0.3	3.8482	0.6451
0.6	3.1431	1.0083
0.8	2.7627	1.458
1.1	2.5485	1.6935
1.4	2.0574	1.6721
2.0	2.0362	2.4555
2.7	1.7899	2.5203
4.0	1.047	2.258
5.3	1.2199	2.9733

Titration of Py-NO with **2a** (4.4 mM, DMSO-d6).

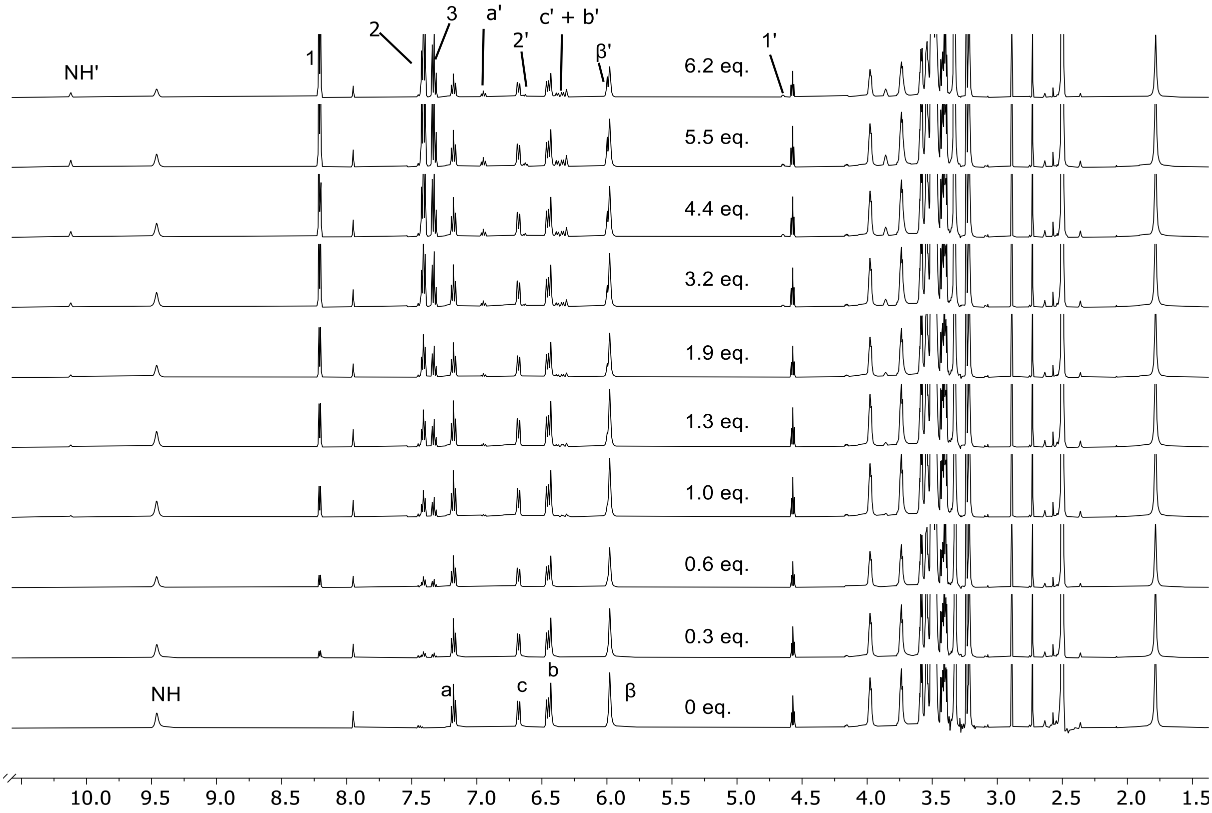

Figure S30. Titration of Py-NO with **2a** (4.4 mM, DMSO-d6).

Table S4. Integrals of titration between **2a** and Py-NO.

Equivalents of guest	Integral of free NH / 9.41 ppm	Integral of bound NH / 10.00 ppm
0	3.5485	0
0.3	4.2625	0.1006
0.6	4.0449	0.2308
1.0	3.8865	0.3445
1.3	3.9016	0.4576
1.9	3.7317	0.5523
3.2	3.8854	0.8171
4.4	3.5868	0.9279
5.5	3.1992	1.1222
6.2	3.1837	1.1041

Titration of Py-NO with **1b** (10.8 mM, DMSO-d_6).

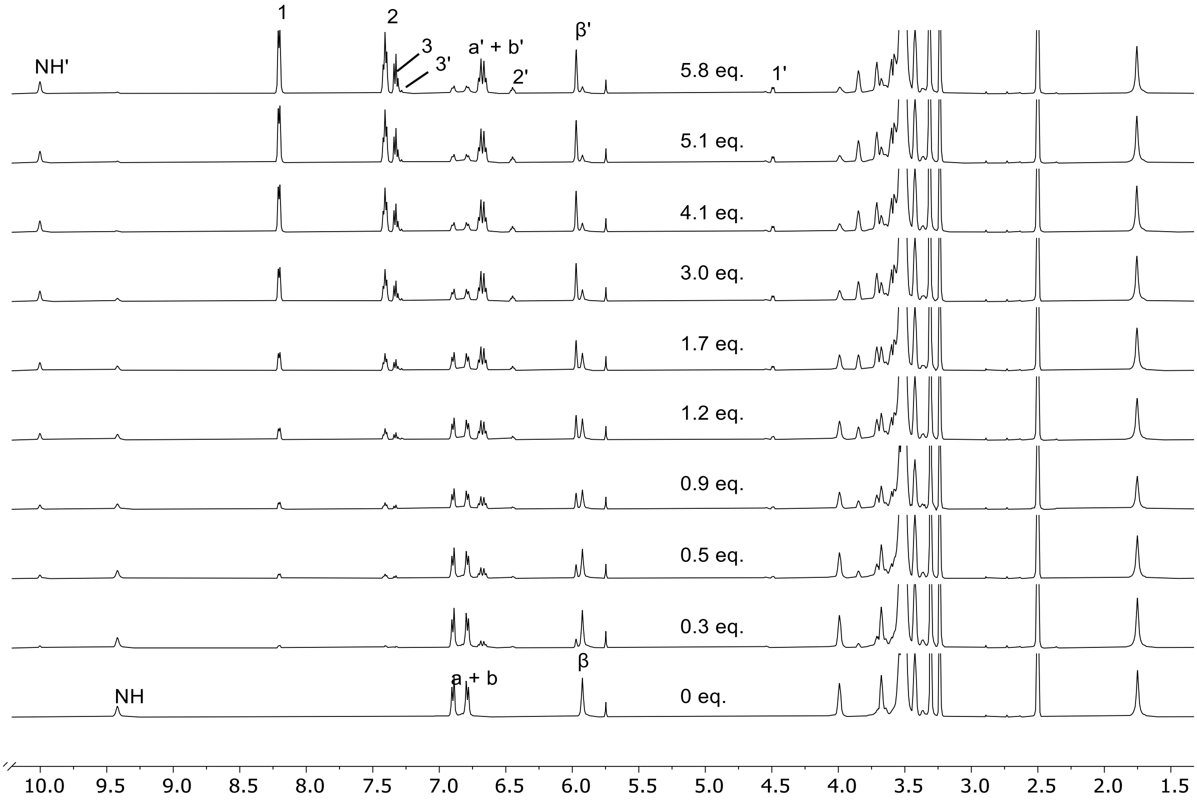

Figure S31. Titration of Py-NO with **1b** (10.8 mM, DMSO-d_6).

Table S5. Integrals of titration between **1b** and Py-NO.

Equivalents of guest	Integral of free NH / 9.42 ppm	Integral of bound NH / 10.00 ppm
0	3.2953	0
0.3	2.7235	0.2181
0.5	2.3564	0.6588
0.9	1.9174	1.0712
1.2	1.7066	1.4151
1.7	1.3919	1.7847
3.0	0.8519	2.3099
4.1	0.6624	2.6298
5.1	0.4801	2.5131
5.8	0.3764	2.5753

Titration of Phe-Py-NO with **1b** (9.8 mM, DMSO-d_6).

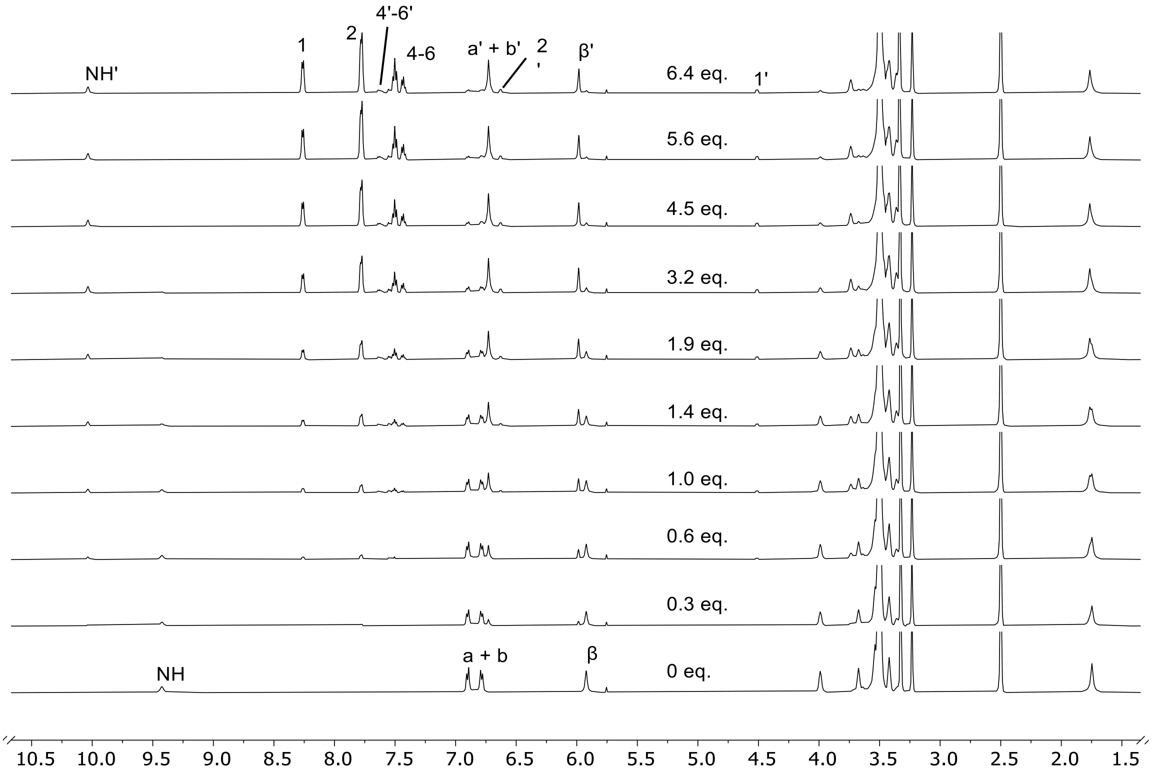

Figure S32. Titration of Phe-Py-NO with **1b** (9.8 mM, DMSO-d_6).

Table S6. Integrals of titration between **1b** and Phe-Py-NO.

Equivalents of guest	Integral of free NH / 9.42 ppm	Integral of bound NH / 10 ppm
0	3.2148	0
0.3	2.7377	0.4011
0.6	2.2513	0.8199
1.0	1.8777	1.2721
1.4	1.5275	1.6672
1.9	1.1774	2.0998
3.2	0.6366	2.7059
4.5	0.5478	3.1783
5.6	0.3928	3.324
6.4	0.3602	3.2304

Titration of Py-NO with **1c** (8.1 mM, D_2O).

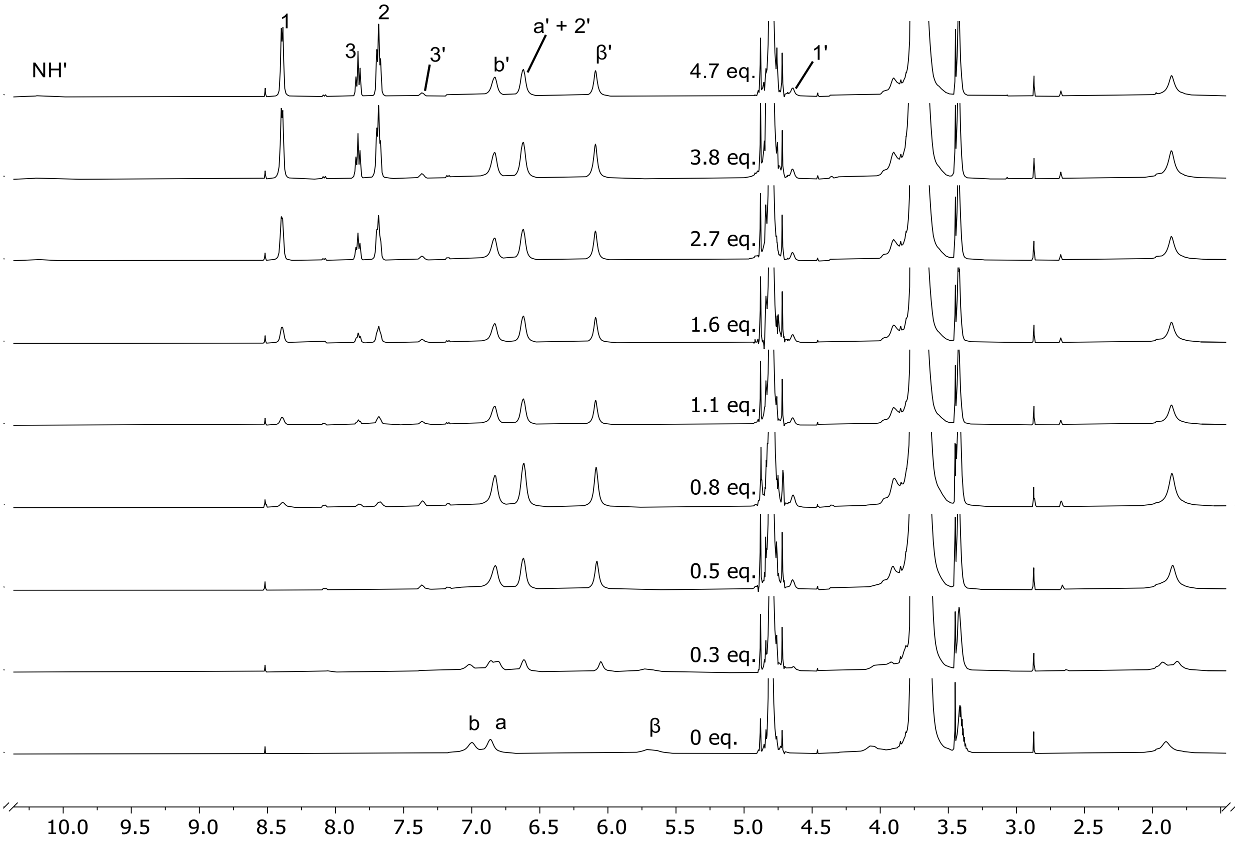

Figure S33. Titration of Py-NO with **1c** (8.1 mM, D_2O).

Titration of Phe-Py-NO with **1c** (6.0 mM, D_2O).

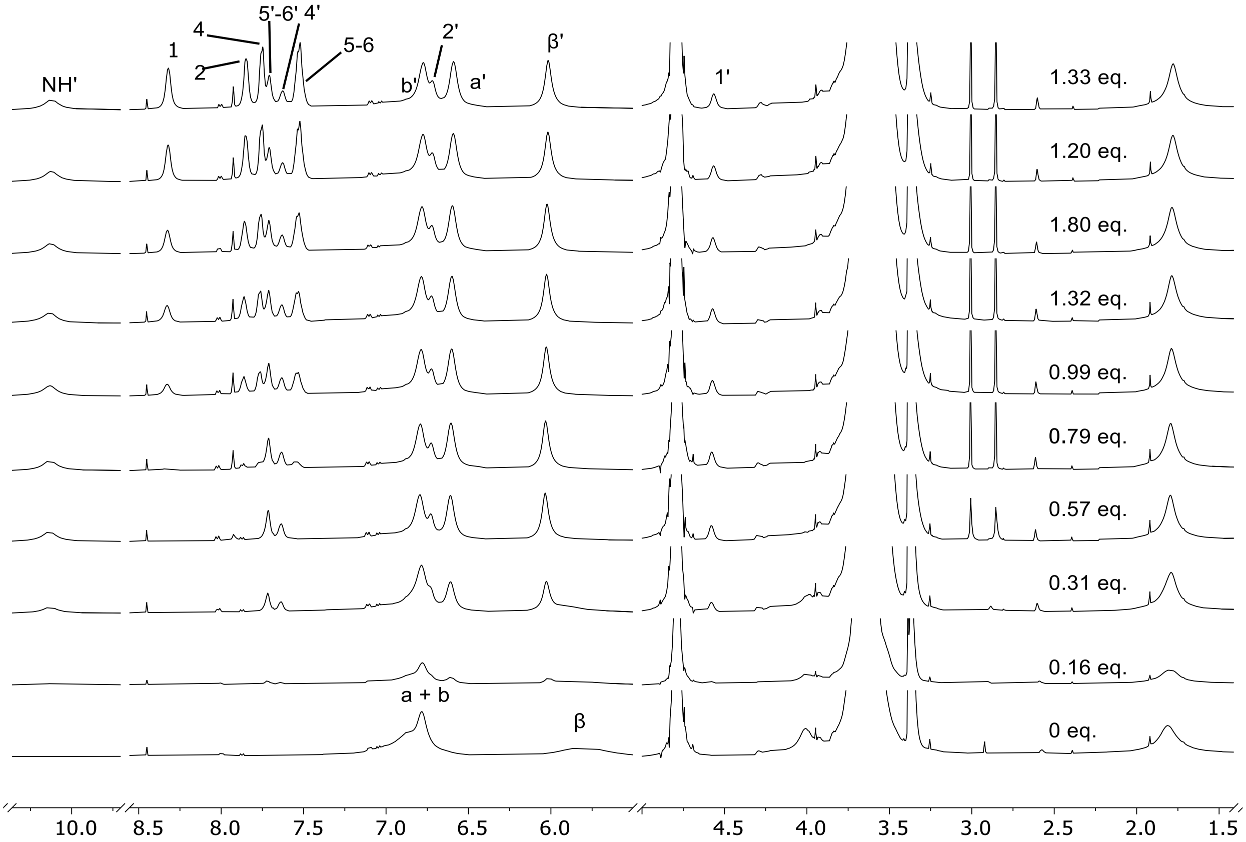

Figure S34. Titration of Phe-Py-NO with **1c** (6.0 mM, D_2O).

Titration of Py-NO with Triton X-100 (6.1 mM, D_2O).

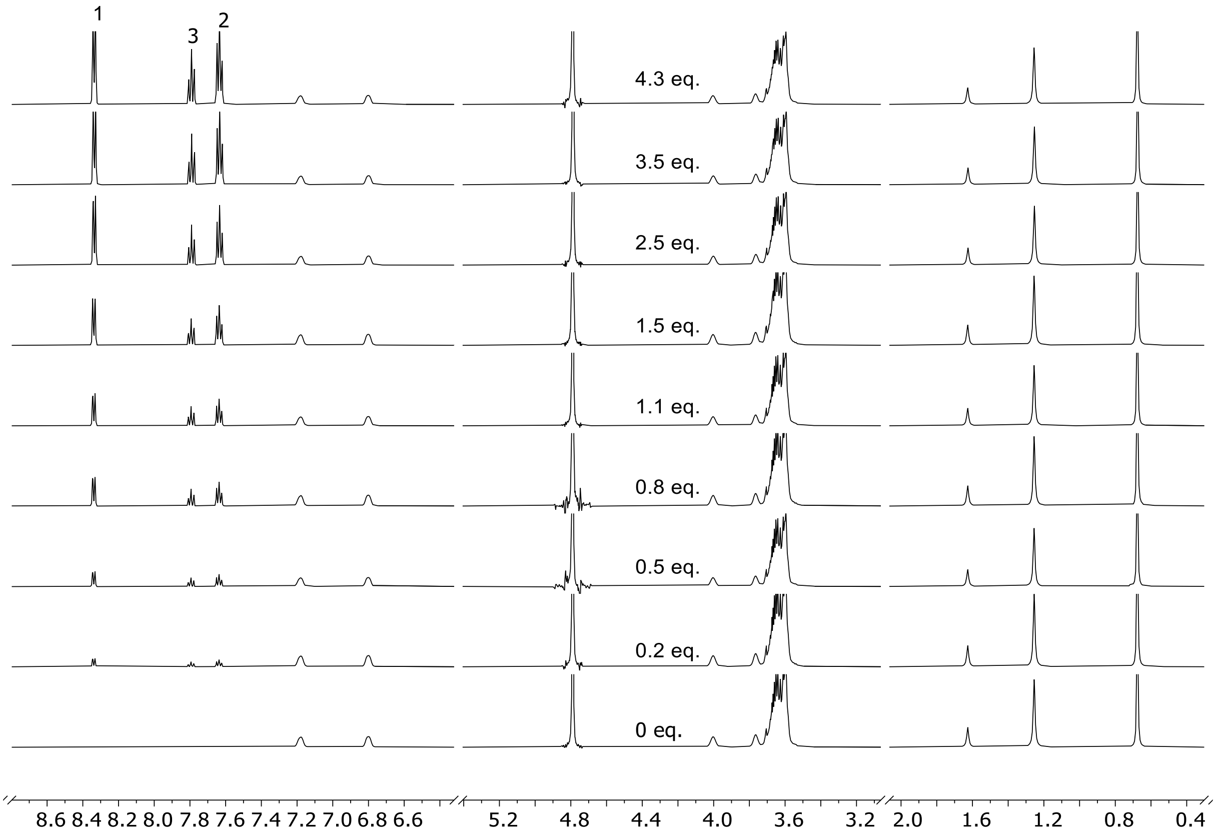

Figure S35. Titration of Py-NO with Triton X-100 (6.1 mM, D_2O).

TX-100 does not exhibit interactions with the guest Py-NO.

Titration of Py-NO with Tergitol (7.4 mM, D_2O).

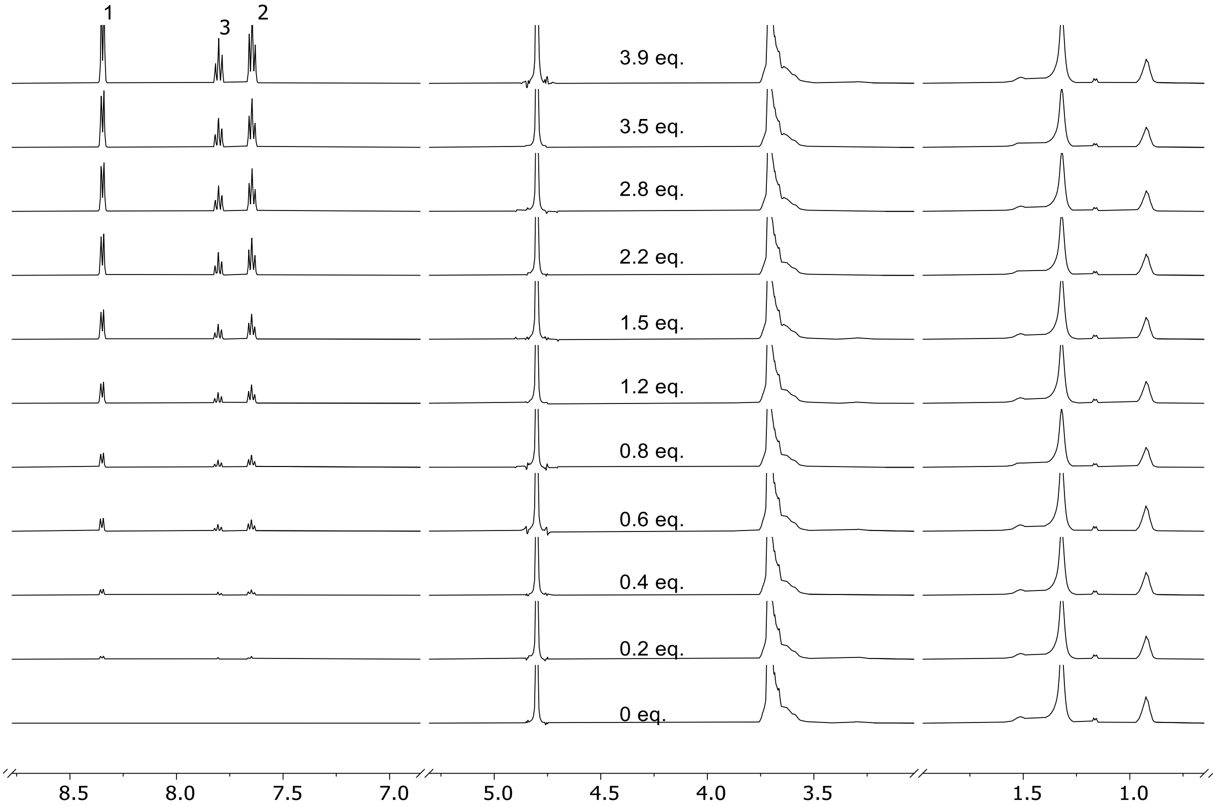

Figure S36. Titration of Py-NO with Tergitol (7.4 mM, D_2O).

Tergitol does not exhibit interactions with the guest Py-NO.

Micellar studies

CMC, cloud point and water solubility

The critical micelle concentration of **1b** and **1c** were determined by diluting an aqueous 6.5 mM stock solution of the corresponding calix[4]pyrrole into water (repeated 1:1 dilution) and measuring the count number using DLS at attenuator position 3 with backscattering detection after each dilution. The CMC of the calix[4]pyrrole was determined to be the concentration at which the count number of the solution corresponded to the count number of pure water, or the point at which the fit lines of the count numbers above and below CMC crossed each other (Figure S37, Figure S38).

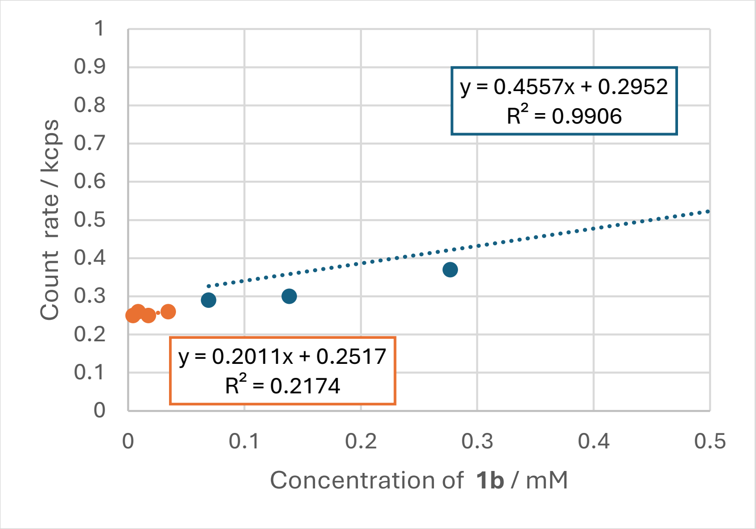

Figure S37. CMC determination of **1b**.

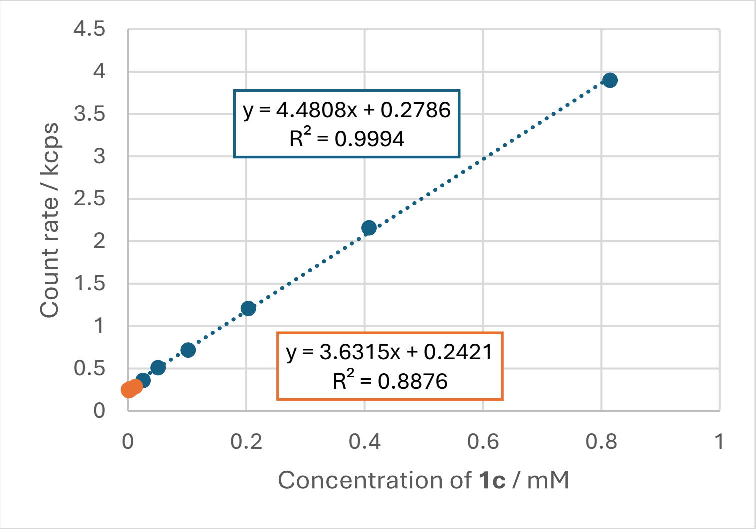

Figure S38. CMC determination of **1c**.

Particle sizes and cloud point of TX-100/calix[4]pyrrole mixed micelles

A representative graph of a single DLS-measurement and its correlograms are shown in Figure S39 and Figure S40, respectively.

For DLS, each sample was measured as a triplicate, and average value for the Zeta-average and polydispersity index are reported (Table S7).

The cloud point temperature was recorded to be the temperature, where turbidity was observed (Table S8).

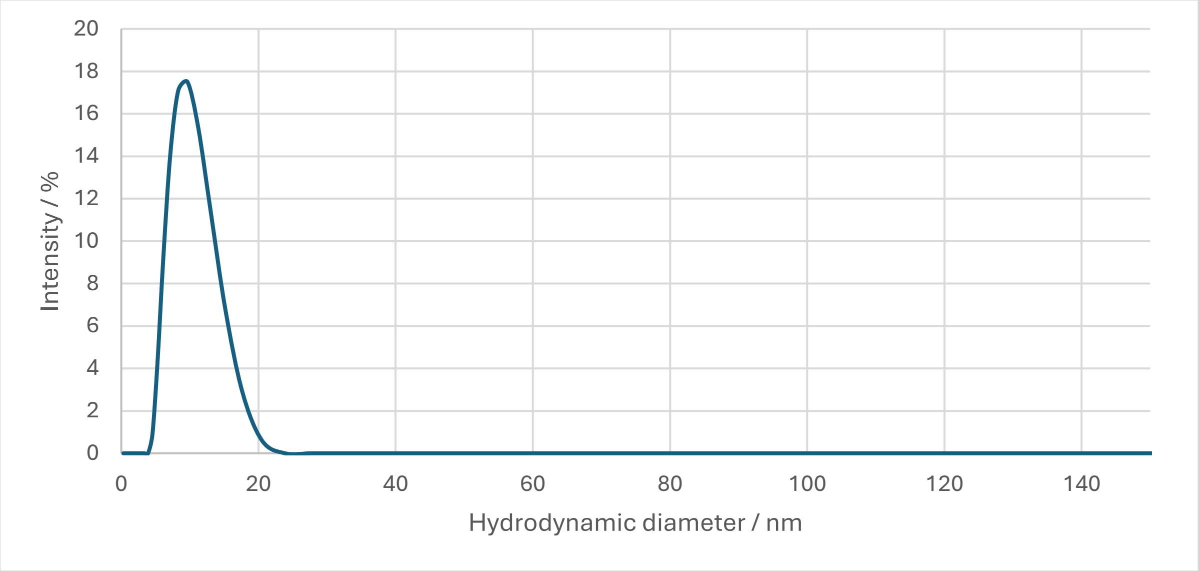

Figure S39. Size distribution graph of Triton X-100 with 3.3% **1a**.

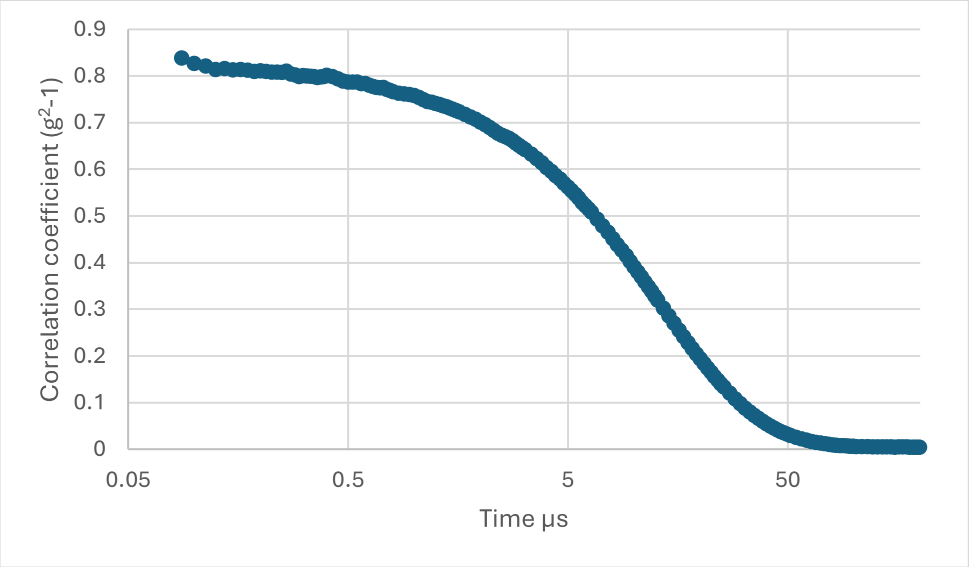

Figure S40. Correlogram of the DLS-measurement of Triton X-100 with 3.3% **1a**.

Table S7. Zeta-averages of the hydrodynamic diameters of 0.97% TX-100 solutions in 3.3% MeOH with added calix[4]pyrroles.

	Percentage calix[4]pyrrole /%	Measurement	Z-average	Polydispersity (PD)	Average Z-average	Average PD	Z-average standard deviation
control	0	1	8.624	0.0829	8.63	0.08	0.04
		2	8.673	0.06509			
		3	8.584	0.08506			
**1a**	0.65	1	8.997	0.1031	8.93	0.11	0.09
		2	8.821	0.08667			
		3	8.97	0.1282			
	0.98	1	8.725	0.08878	8.65	0.08	0.07
		2	8.608	0.06798			
		3	8.607	0.07262			
	1.96	1	8.894	0.09865	8.82	0.11	0.08
		2	8.744	0.09321			
		3	8.82	0.1295			
	3.27	1	9.039	0.1519	9.23	0.15	0.17
		2	9.374	0.1403			
		3	9.265	0.1501			
	4.25	1	9.12	0.1216	9.02	0.12	0.15
		2	8.849	0.11			
		3	9.082	0.1142			
	5.56	1	9.299	0.1288	9.19	0.11	0.10
		2	9.138	0.09806			
		3	9.119	0.1055			
	6.54	1	9.301	0.1513	9.24	0.13	0.06
		2	9.181	0.1113			
		3	9.239	0.1182			
**2a**	0.61	1	8.7	0.14	8.70	0.09	0.05
		2	8.754	0.0719			
		3	8.651	0.04974			
	0.92	1	8.302	0.044	8.55	0.05	0.22
		2	8.687	0.04923			
		3	8.661	0.05843			
	1.84	1	8.917	0.07971	9.12	0.11	0.19
		2	9.287	0.1268			
		3	9.161	0.1245			
	3.07	1	9.069	0.09924	9.10	0.12	0.10
		2	9.210	0.1794			
		3	9.016	0.06955			
	4.00	1	9.419	0.09635	9.43	0.11	0.03

		2	9.401	0.15			
		3	9.463	0.1324			
	5.23	1	9.616	0.1356	9.51	0.12	0.12
		2	9.373	0.1009			
		3	9.526	0.1277			
	6.15	1	9.754	0.09982	9.69	0.10	0.0.7
		2	9.69	0.1084			
		3	9.615	0.08853			
**1c**	0.63	1	8.886	0.1485	9.03	0.20	0.13
		2	9.142	0.2266			
		3	9.051	0.2304			
	0.95	1	8.413	0.1914	8.68	0.12	0.37
		2	8.512	0.07995			
		3	9.101	0.09445			
	1.90	1	8.66	0.0907	8.64	0.10	0.10
		2	8.732	0.1444			
		3	8.536	0.05807			
	3.16	1	8.292	0.1059	8.27	0.13	0.11
		2	8.368	0.1974			
		3	8.153	0.07435			
	4.11	1	24.9	0.1316	8.29	0.18	0.11
		2	8.21	0.171			
		3	8.372	0.2452			
	5.37	1	8.266	0.2137	8.39	0.19	0.48
		2	8.92	0.1424			
		3	7.988	0.2285			
	6.32	1	8.561	0.1332	8.32	0.18	0.22
		2	8.257	0.2215			
		3	8.139	0.198			
**2**	0.65	1	9.132	0.07161	9.07	0.06	0.06
		2	9.047	0.05471			
		3	9.024	0.05991			
	0.98	1	9.424	0.1071	9.37	0.12	0.11
		2	9.239	0.07896			
		3	9.435	0.1729			
	1.96	1	10.36	0.1235	10.33	0.17	0.04
		2	10.29	0.2168			
		3	10.34	0.1708			
	3.26	1	11.95	0.1582	11.74	0.17	0.19
		2	11.57	0.1712			
		3	11.7	0.1671			

	4.24	1	12.21	0.1002	12.26	0.10	0.04
		2	12.27	0.09734			
		3	12.29	0.09088			
	5.55	1	14.25	0.1516	14.60	0.16	0.63
		2	14.23	0.1553			
		3	15.33	0.1627			
	6.52	1	16.13	0.1646	15.94	0.15	0.17
		2	15.8	0.1321			
		3	15.9	0.1455			
**1**	0.67	1	9.451	0.1484	9.32	0.11	0.12
		2	9.243	0.09613			
		3	9.26	0.08923			
	1.01	1	9.501	0.1154	9.41	0.11	0.08
		2	9.333	0.07858			
		3	9.408	0.1272			
	2.01	1	10.08	0.06822	10.04	0.08	0.06
		2	10.07	0.09058			
		3	9.971	0.08749			
	3.35	1	11.85	0.09131	11.73	0.09	0.12
		2	11.71	0.09373			
		3	11.62	0.07538			
	4.36	1	12.71	0.1135	12.69	0.10	0.02
		2	12.68	0.08522			
		3	12.69	0.115			
	5.70	1	14.63	0.103	14.58	0.11	0.08
		2	14.49	0.1099			
		3	14.63	0.1143			
	6.71	1	16.75	0.09878	16.59	0.11	0.17
		2	16.62	0.1163			
		3	16.41	0.1086			
**1b**	0.54	1	8.806	0.1987	8.69	0.19	0.15
		2	8.74	0.244			
		3	8.524	0.1396			
	0.81	1	8.561	0.1307	8.54	0.12	0.02
		2	8.533	0.125			
		3	8.515	0.1032			
	1.61	1	9.873	0.1399	8.82	0.15	0.92
		2	8.343	0.1996			
		3	8.23	0.1112			
	2.69	1	8.62	0.2574	8.49	0.24	0.14
		2	8.34	0.2268			

		3	8.497	0.2409			
	3.50	1	8.82	0.2176	8.71	0.24	0.11
		2	8.709	0.2463			
		3	8.603	0.2704			
	4.57	1	9.605	0.2755	9.31	0.27	0.52
		2	8.705	0.2771			
		3	9.609	0.2506			
	5.38	1	9.142	0.2637	9.55	0.23	0.61
		2	10.25	0.2383			
		3	9.26	0.1997			

Table S8. Cloud point temperatures of 0.97% TX-100 solutions with added calix[4]pyrroles in 3.3% MeOH.

Calix[4]pyrrole	Concentration / w/V%	Cloud point / °C	Calix[4]pyrrole	Concentration / w/V%	Cloud point / °C
**1**	0	75	**2a**	0	75
	0.67	71		0.61	74
	1.01	72		0.92	72
	2.01	71		1.84	69
	3.35	63		3.07	68
	4.36	60		4.00	65
	5.70	55		5.23	63
	6.71	50		6.15	61
**2**	0	75	**1b**	0	75
	0.65	73		0.54	79
	0.98	74		0.81	78
	1.96	74		1.61	78
	3.26	70		2.69	77
	4.24	69		3.50	78
	5.55	67		4.57	79
	6.52	67		5.38	79
**1a**	0	75	**1c**	0	75
	0.65	72		0.63	74
	0.98	73		0.95	75
	1.96	71		1.90	77
	3.27	64		3.16	79
	4.25	58		4.11	80
	5.56	53		5.37	82
	6.54	51		6.32	83

NOESY of **1c** and Triton X-100

NOESY NMR experiment (Figure S41) was done on a solution of **1c** (5.2 mM) with Triton X-100 (1.3 equivalents) in D_2O.

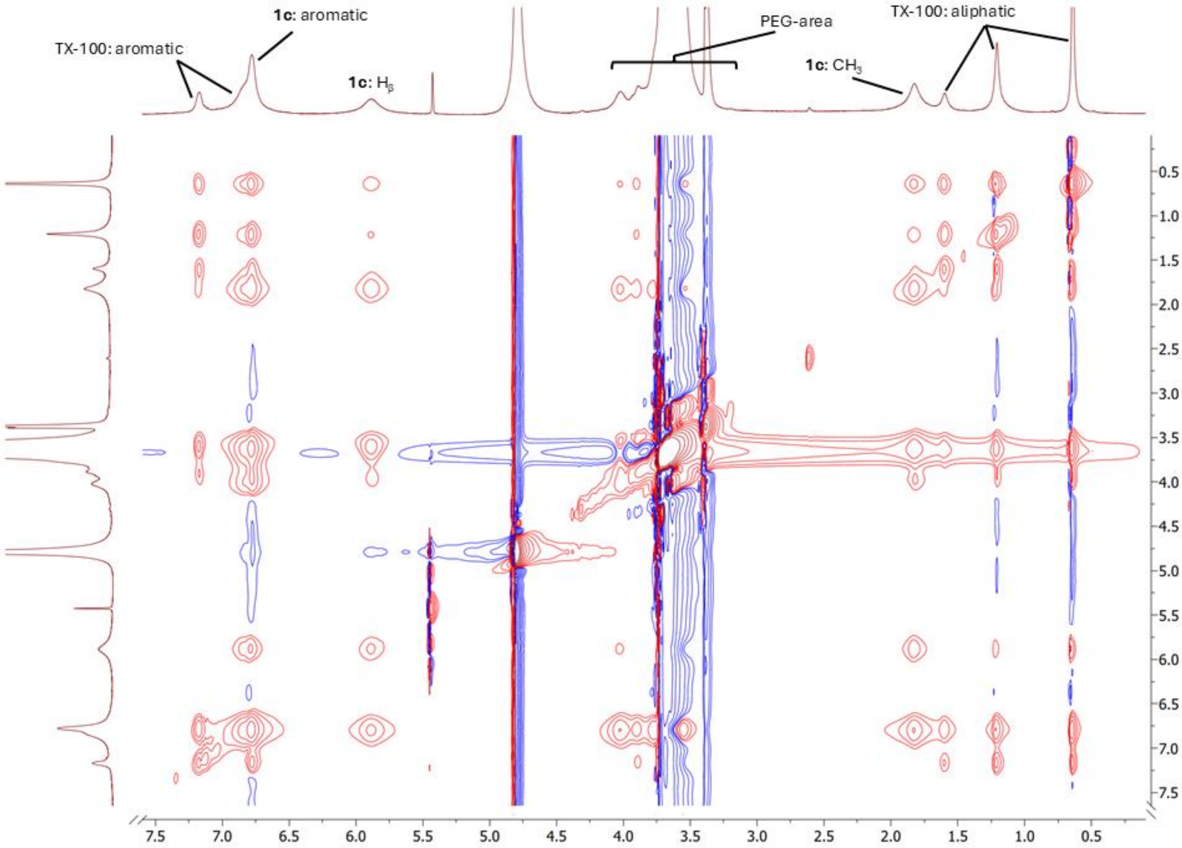

Figure S41. NOESY of **1c** and TX-100.

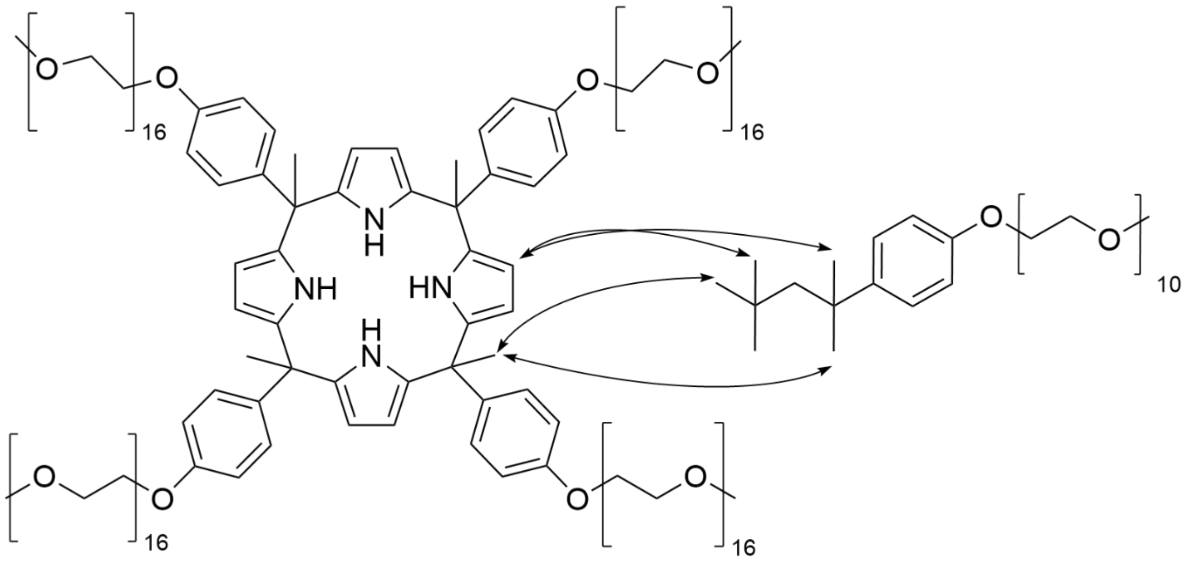

Figure S42. Interactions between **1c** and Triton x-100.

Cloud point extraction of Py-NO with **1c** and surfactant: Examples of chromatograms

Control; no TX-100 or pC4P-900

Control samples were run to determine the elution times of each of the components (Py-NO, Triton X-100 and **1c**). Tergitol 15-S-7 is UV-inactive, so it was not used in the control experiment.

Table S9. Results of control measurements.

	Calibrant / Sample	Absorbance / AU	Conc. / ppm
Calibration solutions	25 ppm	331.934	27.1
	50 ppm	685.941	54.2
	100 ppm	1387.36	108.4
Samples	All	177.806	15.15
	No TX-100	196.313	16.58
	No **1c**	520.352	41.55

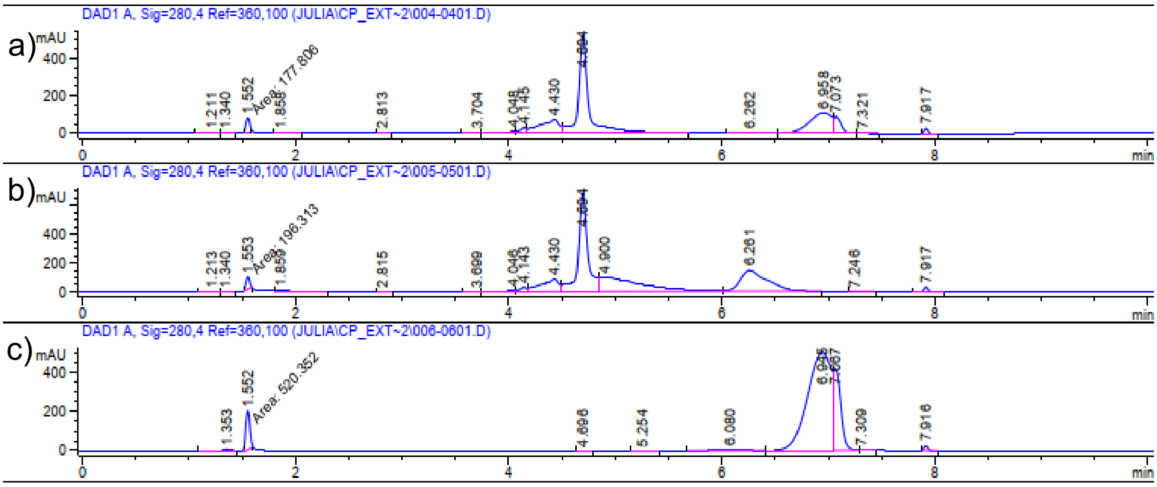

Figure S43. Chromatograms of HPLC control measurements. a) 1.8 eq. **1c** and 2.8% TX-100, b) 1.8 eq. **1c** and 0% TX-100, c) 0 eq. **1c** and 2.8% TX-100.

Variable equivalents of calixpyrrole to Py-NO

Table S10. Results of HPLC analysis of CPE of 0.4 mM Py-NO with 1-5 eq. of **1c** and 2.8% TX-100.

	Calibrant / Sample	Absorbance / AU	Conc. / ppm
Calibration solutions	5 ppm	64.4628	5.00
	10 ppm	130.308	9.99
	25 ppm	314.332	24.98
	50 ppm	633.108	49.95
	100 ppm	1236.17	99.90
Samples	1 eq.	239.324	18.46
	2 eq.	159.996	12.00
	3 eq.	124.899	9.15
	4 eq.	93.8339	6.60
	5 eq.	75.1785	5.08

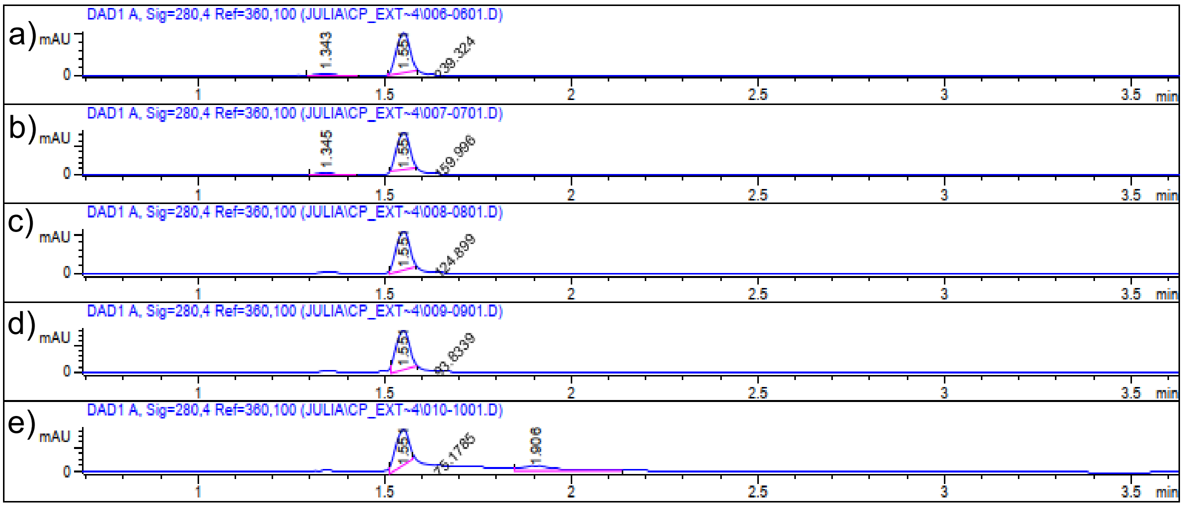

Figure S44. Chromatograms of extraction of 0.4 mM Py-NO with 2.8% TX-100 and a) 1 equivalents, b) 2 eq., c) 3 eq., d) 4 eq. and e) 5 eq. of **1c**.

Variable concentration of TX-100

Table S11. Results of HPLC analysis of CPE of 0.4 mM Py-NO with 2 eq. **1c** and 1-5% of TX-100.

	Calibrant / Sample	Absorbance / AU	Conc. / ppm
Calibration solutions	5 ppm	72.6194	5.00
	10 ppm	134.406	9.99
	25 ppm	331.176	24.98
	50 ppm	648.617	49.95
	100 ppm	1261.75	99.90
Samples	1%	123.031	7.94
	2%	143.947	9.62
	3%	151.685	10.25
	4%	140.711	9.36
	5%	141.401	9.42

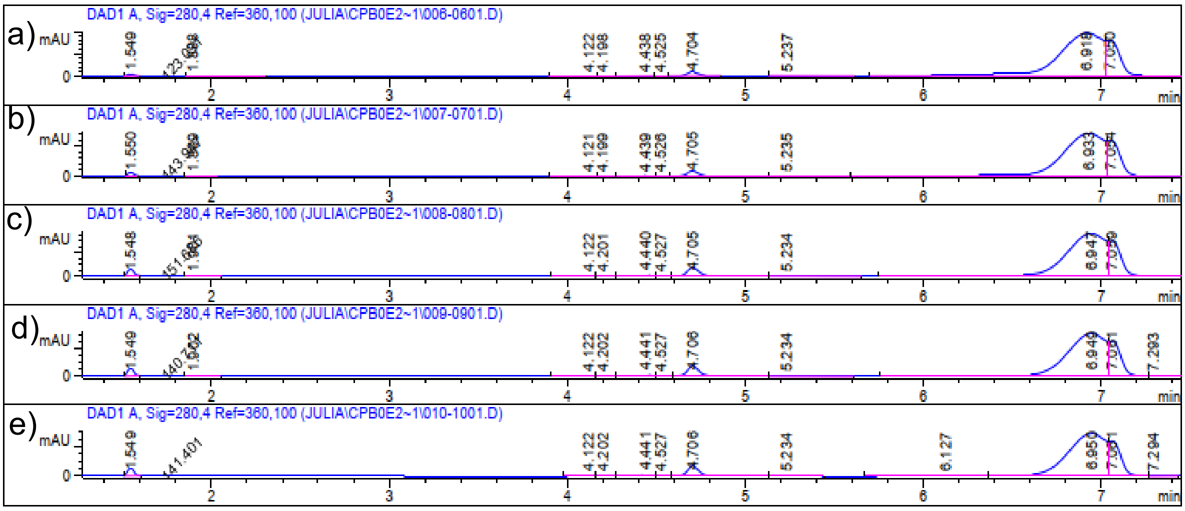

Figure S45. Chromatograms of CPE of 0.4 mM Py-NO with 2 eq. **1c** and a) 1%, b) 2%, c) 3%, d) 4% and e) 5% of TX-100.

Variable concentration of Tergitol 15-S-7

Table S12. Results of HPLC analysis of CPE of 0.4 mM Py-NO with 2 eq. **1c** and 1-5% of Tergitol.

	Calibrant / Sample	Absorbance / AU	Conc. / ppm
Calibration solutions	5 ppm	62.6642	45.00
	10 ppm	134.495	9.99
	25 ppm	327.698	24.98
	50 ppm	651.092	49.95
	100 ppm	1269.43	99.90
Samples	1%	164.196	12.35
	2%	162.896	12.25
	3%	144.826	10.83
	4%	131.65	9.79
	5%	132.253	9.84

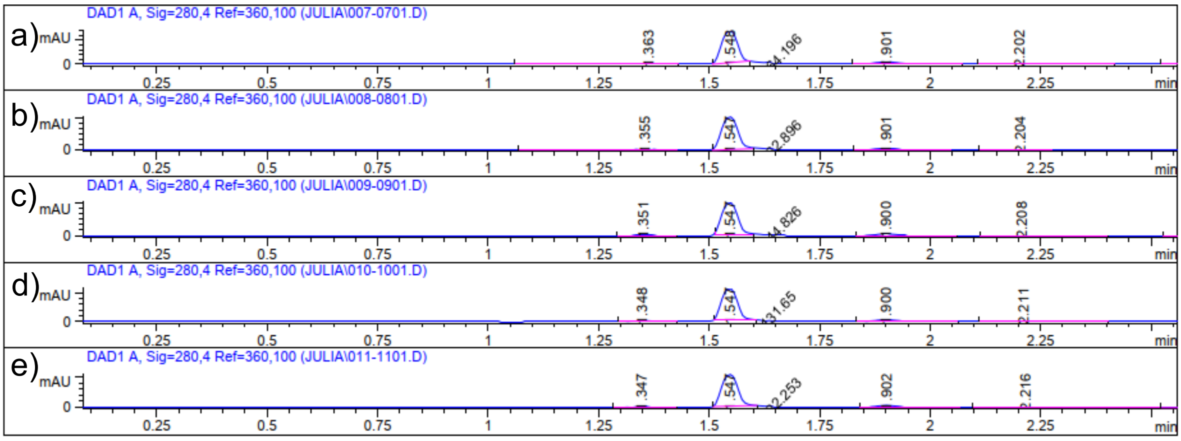

Figure S46. Chromatograms of CPE of 0.4 mM Py-NO with 2 eq. **1c** and a) 1%, b) 2%, c) 3%, d) 4% and e) 5% of Tergitol 15-S-7.

Results

Table S13. Extraction efficiencies of Py-NO with 2 equivalents **1c** and varying percentage of Triton X-100.

Percentage surfactant / %	Measured concentration of Py-NO / ppm			Extraction efficiency / %			Average extraction efficiency / %	Standard deviation
	Trial 1	2	3	Trial 1	2	3		
0	40.82	41.69	41.05	4.37	2.32	3.84	3.5	1.1
1	7.94	15.14	9.22	79.74	61.35	76.46	72.5	9.8
2	9.64	14.63	15.62	75.44	62.66	60.14	66.1	8.2
3	10.25	12.86	17.12	73.84	67.18	56.31	65.8	8.9
4	9.36	14.41	10.32	76.10	63.22	73.66	71.0	6.8
5	9.42	13.75	14.90	79.96	64.90	61.98	67.6	7.4

Table S14. Extraction efficiencies of Py-NO with 2.8% Triton X-100 and varying equivalents of **1c**.

Equivalents **1c**	Measured concentration of Py-NO / ppm			Extraction efficiency / %			Average extraction efficiency / %	Standard deviation
	Trial 1	2	3	Trial 1	2	3		
0	41.55	34.57	34.59	2.66	11.78	11.71	8.7	5.2
1	18.46	20.99	19.37	52.88	46.43	50.56	50.0	3.3
2	12.00	14.31	12.04	69.38	63.49	69.27	67.4	3.4
3	9.14	10.16	9.38	76.68	74.06	76.06	75.6	1.4
4	6.60	6.32	6.87	83.15	83.87	82.46	83.2	0.7
5	5.08	7.23	5.72	87.03	81.56	85.41	84.7	2.8

Table S15. Extraction efficiencies of Py-NO with 2 equivalents of **1c** and varying equivalents of Tergitol 15-S-7.

Percentage surfactant / %	Measured concentration of Py-NO / ppm			Extraction efficiency / %			Average extraction efficiency / %	Standard deviation
	Trial 1	2	3	Trial 1	2	3		
1	12.35	20.54	20.98	68.47	47.57	46.46	54.2	12.4
2	12.25	11.72	11.70	68.73	70.08	70.13	69.6	0.8
3	10.83	10.83	10.83	72.37	72.36	72.35	72.36	0.01
4	9.79	10.12	10.20	75.02	74.17	73.97	74.4	0.6
5	9.84	8.97	10.09	74.90	77.11	74.24	75.4	1.5

Table S16. Extraction efficiencies of Py-NO with 2.7% Tergitol 15-S-7 and varying equivalents of **1c**.

Equivalents **1c**	Measured concentration of Py-NO / ppm			Extraction efficiency / %			Average extraction efficiency / %	Standard deviation
	Trial 1	2	3	Trial 1	2	3		
0	40.02	39.64	40.97	-2.15	-1.16	-4.57	-2.6	1.8
1	14.74	16.14	16.13	62.37	58.82	58.84	60.0	2.0
2	9.89	9.061	9.39	74.76	76.87	76.03	75.9	1.1
3	6.70	6.61	8.49	82.90	83.13	78.32	81.5	2.7
4	7.28	8.99	8.98	81.43	77.04	77.08	78.5	2.5
5	1.86	3.31	3.35	95.25	91.54	91.45	92.7	2.2

References

1 P. Anzenbacher, K. Jursíková, V. M. Lynch, P. A. Gale and J. L. Sessler, *J. Am. Chem. Soc.*, 1999, **121**, 11020–11021.

2 L. Bonomo, E. Solari, G. Toraman, R. Scopelliti, M. Latronico and C. Floriani, *Chem. Commun.*, 1999, 2413–2414.

3 M. Pamuła, E. Bulatov and K. Helttunen, *J. Mol. Struct.*, 2023, **1273**, 134268.

4 K. D. Bhatt, H. D. Shah, K. M. Modi, M. B. Narechania and C. Patel, *Supramol. Chem.*, 2019, **31**, 268–282.

5 M. Ouchi, Y. Inoue, Y. Liu, S. Nagamune, S. Nakamura, K. Wada and T. Hakushi, *Bull. Chem. Soc. Jpn.*, 1990, **63**, 1260–1262.

6 R. Nguyen, N. Jouault, S. Zanirati, M. Rawiso, L. Allouche, G. Fuks, E. Buhler and N. Giuseppone, *Soft Matter*, 2014, **10**, 2926–2937.
